# Pinpointing genomic regions conferring herbicide tolerance in cassava via genome-wide association mapping

**DOI:** 10.1007/s11103-026-01761-3

**Published:** 2026-09-09

**Authors:** Reisane Teles Santiago, Massaine Bandeira e Souza, Diego Fernando Marmolejo Cortes, Eder Jorge de Oliveira

**Affiliations:** 1 Universidade Federal do Recôncavo da Bahia, Campus Cruz das Almas, Cruz das Almas, 44380-000 BA Brazil; 2 Embrapa Mandioca e Fruticultura, Nugene, Cruz das Almas, 44380-000 BA Brazil

**Keywords:** *Manihot esculenta* Crantz, GWAS, Detoxification, SNPs, Redox metabolism

## Abstract

Cassava (*Manihot esculenta* Crantz) is a tropical crop of major socioeconomic importance, whose productivity can be limited by sensitivity to herbicides used for weed management. This study aimed to perform a genome-wide association study (GWAS) in 194 cassava genotypes to identify genomic regions associated with tolerance to the herbicides mesotrione, S-metolachlor, and chloransulam-methyl. The evaluations performed at 3, 6, 9, 15, and 30 days after application (DAA) were used to characterize the temporal progression of phytotoxicity. Based on this analysis, the phenotype obtained at 9 days after application (PhytoX9DAA) was selected for genome-wide association analyses because it represented the period of greatest symptom expression and the highest discrimination among genotypes. GWAS analyses were performed using de-regressed BLUPs and the MLM, MLMM, and BLINK models, incorporating kinship (K) and population structure (Q) matrices. Significant markers were detected across multiple chromosomes, and the corresponding genomic windows contained candidate genes with functional annotations related to herbicide response. The predominant functional categories included membrane transport, channel activity, signal peptide processing, protein phosphorylation, cellular signaling, and metabolic regulation. Key candidate genes included Manes.02G151900 and Manes.02G152700 (chromosome 2), associated with transmembrane transport and signal peptide processing; Manes.09G060900 (chromosome 9), associated with protein kinase activity, ATP binding, and protein phosphorylation; and Manes.15G083800 and Manes.15G084000 (chromosome 15), associated with S-adenosylmethionine-dependent methyltransferase activity, membrane-related functions, and protein phosphorylation. These genes participate in biochemical pathways involved in cellular signaling, membrane transport, and metabolic regulation that may contribute to herbicide tolerance. Overall, the results demonstrate that herbicide tolerance in cassava is a quantitative and polygenic trait governed by numerous small-effect loci. The integration of cellular signaling, metabolic regulation, and membrane transport supports the physiological resilience of the species under chemical exposure, providing valuable insights for breeding strategies and marker-assisted selection.

**Supplementary Information:**

The online version contains supplementary material available at https://doi.org/10.1007/s11103-026-01761-3.

## Key Message

The first genomic dissection of herbicide tolerance in cassava revealed herbicide-specific genetic architectures and candidate loci for mesotrione and S-metolachlor tolerance.

**Supplementary Information:**

The online version contains supplementary material available at https://doi.org/10.1007/s11103-026-01761-3.

## Introduction

Cassava (*Manihot esculenta* Crantz) is a crop of major importance in tropical and subtropical regions, playing a central role in food security and in the livelihoods of millions of people who depend on it as both a staple food and a source of income (Amelework et al. 2021). Its broad adaptability to diverse edaphoclimatic conditions, combined with the growing economic relevance of cassava-based value chains (flour, starch, and other derivatives), reinforces its potential as a globally important crop (Otekunrin 2024). In addition to producing energy-rich storage roots, cassava is notable for the nutritional value of its leaves—rich in proteins, vitamins, and minerals—and for its capacity to thrive in low-fertility soils and under water-limited conditions. These characteristics make cassava a strategic crop for vulnerable regions and an important source of employment when effectively integrated into production systems and markets (Borku 2025).

In Brazil, cassava holds strong socioeconomic importance across different levels of agricultural intensification. However, increasing production costs, particularly those associated with weed control, have recently constrained the expansion of cassava cultivation. To address this challenge, there has been a gradual increase in the adoption of herbicides registered for the crop, such as clomazone, ametryn, and S-metolachlor. This practice has become an important complementary strategy to improve weed management efficiency and enhance crop productivity and competitiveness (Costa et al. 2020). In other regions, including African countries such as Nigeria and Tanzania, the use of pre- and post-emergence herbicides in cassava has been evaluated under field conditions. These studies demonstrated effective weed control, reduced dependence on manual weeding, and increased root yield (Ekeleme et al. 2020, 2021; Leonard et al. 2023). Consequently, the adoption of selective herbicides has emerged as a key strategy to reduce interspecific competition, optimize resource use, and decrease the labor burden associated with weed management (Ekeleme et al. 2020; Leonard et al. 2023).

Despite advances in weed management practices, the genetic tolerance of cassava to herbicides remains poorly explored, particularly regarding the genetic architecture underlying this trait. Early studies reported variation in herbicide tolerance among cassava cultivars. For instance, Silveira et al. (2012) observed visible injury symptoms following mesotrione application at different doses in five cassava cultivars, although no significant effects were detected on plant height, stem diameter, leaf number, or dry matter accumulation. In another study, the cultivar Aciolina showed tolerance to the herbicides haloxyfop, quizalofop, and chlorimuron-ethyl but exhibited high sensitivity to imazethapyr and to combinations of lactofen + quizalofop-P-ethyl (Bandeira et al. 2016). Other investigations reported that herbicides such as sulfentrazone and glyphosate caused negative effects in cassava, whereas others—including clomazone, fomesafen, and fluazifop-p-butyl—exhibited low toxicity and did not affect chlorophyll fluorescence parameters (Fv/Fm) or electron transport rate (ETR) (Ferreira et al. 2015).

From a physiological perspective, herbicide tolerance may involve target-site resistance mechanisms or non-target-site resistance mechanisms, including oxidative metabolism mediated by cytochrome P450 enzymes, conjugation via glutathione S-transferases (GSTs), and vacuolar sequestration through ABC (ATP-binding cassette) transporters—processes widely documented in both crop and weed species (Délye 2012; Yu and Powles 2014). Because metabolic resistance typically represents a quantitative and dynamic process, often arising from the accumulation of multiple mechanisms, variation in the activity of these detoxification systems may modulate the phenotypic response of plants to herbicide exposure (Délye 2012; Yu and Powles 2014).

More recently, a transcriptomic study involving 262 cassava varieties identified differentially expressed genes associated with glyphosate resistance, including members of the cytochrome P450, glutathione S-transferase (GST), glycosyltransferase (GT), and ABC transporter families, as well as stress-response genes such as MIOX1, HSP26, and UGT73C5 (Wang et al. 2024). These mechanisms suggest that cassava may partially overcome glyphosate inhibition of the shikimate pathway by activating alternative pathways for aromatic amino acid biosynthesis and enhancing antioxidant capacity. Such findings indicate that herbicide tolerance in cassava involves not only morpho-anatomical and physiological adaptations—such as thickening of the leaf midrib, modifications in vascular tissues, or regrowth following post-emergence herbicide application (Santos et al. 2022a), but also molecular resistance mechanisms, including herbicide metabolism, transport, and detoxification processes.

The expression of herbicide tolerance may also be influenced by environmental factors such as temperature, humidity, and water availability, which affect herbicide absorption, translocation, and metabolism within plants, thereby influencing herbicide efficacy and selectivity (Kumar et al. 2023). Consequently, genotype × environment interactions represent a critical component in interpreting herbicide tolerance phenotypes under field conditions.

From a genomic perspective, genome-wide association studies (GWAS) have emerged as powerful tools for identifying loci and candidate genes associated with complex traits in plant species. GWAS exploits linkage disequilibrium in diverse populations to associate single-nucleotide polymorphisms (SNPs) with phenotypic variation at high resolution (Flint-Garcia et al. 2003; Abou-Khater et al. 2022). However, the robustness of detected associations depends on the genetic diversity of the analyzed panel and on appropriate control of population structure and kinship, since failure to account for these factors can lead to spurious associations (Flint-Garcia et al. 2003; Kang et al. 2010). In rice, GWAS has successfully identified six candidate genes associated with tolerance to glufosinate, glyphosate, and mesotrione through the analysis of 421 cultivars (Xu et al. 2024). A recent review also emphasized that herbicide tolerance has transformed modern agriculture, highlighting the importance of integrated weed management strategies and the development of cultivars with multiple herbicide tolerances (Sharma and Jadhav 2024).

In cassava, GWAS has been used to characterize the genetic architecture of several traits, including root texture (Uchendu et al. 2021), starch pasting properties (Phumichai et al. 2022; Santos et al. 2022b), resistance to African cassava mosaic virus (Wolfe et al. 2016), cassava green mite (Ezenwaka et al. 2018) and cassava brown streak virus (Kayondo et al. 2018). Despite the increasing availability of genomic studies in cassava, no GWAS investigations specifically addressing herbicide tolerance have been reported to date. Addressing this knowledge gap through GWAS could provide valuable insights into the genetic basis of herbicide tolerance in cassava, with important implications for breeding programs, crop sustainability, and efficient weed management.

Given this context, the present study aimed to explore the genetic variability underlying herbicide tolerance in cassava genotypes using GWAS, with the objective of identifying genomic regions and candidate genes involved in detoxification, transport, or regulation of chemical stress responses. To achieve this, an association mapping analysis was conducted in 194 cassava genotypes, focusing on tolerance to the herbicides mesotrione, S-metolachlor, and chloransulam-methyl. The findings are expected to provide valuable insights for cassava improvement programs, including applications in marker-assisted selection and future gene-editing strategies.

## Materials and methods

### Plant material and experimental conditions

A total of 194 cassava genotypes from the Embrapa Cassava and Fruits breeding program (Cruz das Almas, Bahia, Brazil; 12°40′19″S, 39°06′22″W; altitude 226 m) were evaluated (Table S1). The panel consisted predominantly of Brazilian genotypes (94.3%), with smaller contributions from Colombia (5.2%) and Nigeria (0.5%). Regarding genetic origin, 80.9% of the materials were classified as landraces, whereas 19.1% corresponded to improved cultivars (Table S1). Among the Brazilian genotypes, the Northeast region accounted for the largest proportion (60.8%), followed by the North, Southeast, South, and Central-West regions, reflecting the broad diversity of cassava cultivated across Brazil.

Field trials were conducted in an isolated experimental field at the Federal University of Recôncavo da Bahia (UFRB), Cruz das Almas campus (12°40′12″S, 39°06′07″W; altitude 220 m). The regional climate is classified as tropical, with a mean annual temperature of 23.7 °C, average annual precipitation of 1,161 mm, and mean relative humidity of 80.3% (Climate-Data.Org, 2022). Plantings were carried out during April–May 2023 and May 2024, corresponding to the 2023 and 2024 growing seasons, respectively.

Soil preparation followed the standard agronomic practices of the region, including plowing followed by two harrowings and the opening of planting furrows approximately 15 cm deep. Cassava stem cuttings (stakes) were manually placed horizontally at approximately 10 cm depth and subsequently covered with soil. The adopted planting density was 0.90 m between rows and 0.70 m between plants, following recommended cassava management practices for the state of Bahia (Souza et al. 2006).

The experiment was established using an augmented block design comprising 10 blocks and 343 experimental plots, each consisting of six plants according to Santiago (2026). Of the 194 cassava genotypes evaluated, 183 were classified as non-common treatments, whereas 11 served as common treatments (checks): BRS CS-01, BRS Dourada, BRS Kiriris, BRS Novo Horizonte, BRS Poti Branca, Cigana Preta, Corrente, Eucalipto, Vassoura Preta, BRS Mulatinha, and BRS Formosa.

Following preliminary dose–response assays to determine the optimal application rates, three post-emergence herbicides with distinct modes of action were evaluated. Each herbicide was tested in an independent experiment using the same augmented block design and the same panel of 194 cassava genotypes. Mesotrione (commercial formulation Callisto) was applied at 225 g a.i. ha^−1^ and inhibits carotenoid biosynthesis. S-metolachlor (commercial formulation Dual) was applied at 1,920 g a.i. ha^−1^ and inhibits the biosynthesis of very-long-chain fatty acids (VLCFAs). Chloransulam-methyl (commercial formulation Pacto) was applied at 40 g a.i. ha^−1^ and inhibits acetolactate synthase (ALS).

### Herbicide application and phytotoxicity assessment

Herbicide applications were conducted during the 2023 and 2024 growing seasons, always under comparable environmental conditions, with continuous monitoring of temperature, relative humidity, and wind speed, and following the same spraying protocol in both years. For each herbicide, the spray solution was prepared separately immediately before application. Herbicides were applied individually, without tank mixing, and each active ingredient was evaluated in an independent field experiment. In the first experiment (August 2023), air temperature ranged from 21 °C (minimum) to 28 °C (maximum), with an average relative humidity of 95% and wind speed of approximately 5 km h^−1^. In the second experiment (August 2024), temperatures ranged from 20 to 28 °C, with an average relative humidity of 88% and wind speed also close to 5 km h^−1^. In both years, herbicide applications were performed early in the morning on sunny days, using a XP-20 backpack sprayer (Jacto^®^, 20 L) equipped with a twin flat-fan nozzle (model AD/D 110:02). Applications were carried out at a spray volume of 150 L ha^−1^ and a walking speed of 3.0 km h^−1^.

Phytotoxicity was evaluated at 3, 6, 9, 15, and 30 days after herbicide application (DAA). Visual symptoms of phytotoxicity were assessed using a rating scale from 1 to 5 (Santiago 2026), defined as follows: 1 = absence of leaf burn or chlorosis (highly tolerant); 2 = plants showing slight symptoms of leaf burn or chlorosis (tolerant); 3 = plants exhibiting moderate leaf burn or chlorosis (moderately tolerant); 4 = plants with severe leaf burn and chlorosis (susceptible); and 5 = complete leaf burn, stem desiccation, and plant death (highly susceptible). This scale represents ordered categories of increasing injury severity and was designed to provide a standardized assessment of overall phytotoxicity rather than equally spaced quantitative intervals.

Visual scores were converted to percentage injury values using a standardized five-level scale in which scores of 1, 2, 3, 4, and 5 corresponded to 0, 25, 50, 75, and 100% phytotoxicity, respectively, according to the following equation: Phytotoxicity (%) = (Visual score − 1) × 25. Percentage injury values were then used to estimate genotypic best linear unbiased predictors (BLUPs) using linear mixed models. After deregression, the resulting genotypic values were used as phenotypes for genome-wide association analyses (GWAS). No additional data transformation or formal normality test was performed before GWAS, and the deregressed BLUPs were analyzed as continuous quantitative variables. Although the original phytotoxicity scores were recorded on an ordinal five-level scale, their conversion to percentage injury values before BLUP estimation yielded deregressed BLUPs that represent continuous genetic predictions rather than raw ordinal observations, an approach widely adopted in quantitative genetic analyses of visually scored traits.

Phytotoxicity assessments at 3, 6, 9, 15, and 30 DAA were initially used to characterize the temporal dynamics of genotype responses to herbicide application. Symptom progression over time identified the period of maximum phytotoxicity expression. Based on this analysis, deregressed genotypic values obtained at 9 DAA (PhytoX9DAA) were selected for GWAS because they provided the greatest discrimination among genotypes and corresponded to the period of maximum symptom expression.

### DNA extraction and genotyping

Genomic DNA was extracted from young leaves of the cassava diversity panel using the cetyltrimethylammonium bromide (CTAB) protocol described by Doyle and Doyle (1987), with minor modifications to improve DNA purity in tissues rich in polysaccharides and phenolic compounds. DNA integrity was assessed by electrophoresis on 1.0% (w/v) agarose gels stained with ethidium bromide (1.0 mg mL^−1^), by comparing the samples with a Lambda phage DNA concentration standard series (Invitrogen™). DNA samples were subsequently quantified and normalized to a final concentration of 20 ng µL^−1^.

Genotyping was carried out at the Genomic Diversity Facility of Cornell University using the genotyping-by-sequencing (GBS) approach described by Elshire et al. (2011). Briefly, genomic DNA was digested with the ApeKI restriction enzyme and ligated to barcoded adapters to construct multiplexed sequencing libraries. Sequencing was performed on the Illumina Genome Analyzer 2000 platform (Illumina Inc., San Diego, CA, USA).

Raw sequencing data were processed through a pipeline including read quality filtering, alignment to the cassava reference genome (*Manihot esculenta* v7.1), and variant calling using the TASSEL-GBS pipeline (Glaubitz et al. 2014). SNP markers were subjected to quality control by removing those with a genotyping rate lower than 80% (call rate < 0.80) and a minor allele frequency below 5% (MAF < 0.05). Missing genotype data were subsequently imputed using Beagle v4.0 (Browning and Browning 2009). After quality filtering and imputation, a total of 23,047 SNP markers distributed across the 18 cassava chromosomes were retained for downstream analyses. The distribution and density of these markers were evaluated to confirm adequate genomic coverage of the diversity panel.

### Genome-wide association study (GWAS)

For genome-wide association mapping, de-regressed best linear unbiased predictors (BLUPs) derived from phytotoxicity evaluated at 9 days after herbicide application (PhytoX9DAA) were used as phenotypes. This evaluation time was selected based on the temporal progression of symptoms, which identified the period of maximum phytotoxicity expression and provided the greatest discrimination among the evaluated genotypes.

BLUPs were estimated separately for each herbicide and analytical scenario (2023, 2024, and the combined dataset) using linear mixed models fitted with the lmer() function in the lme4 package (Bates et al. 2015). The fitted model was: ${Y}_{ijkl}=\mu+{L}_{i}+{C}_{j}+{B}_{k}+{G}_{l}+(GA{)}_{lm}+{\epsilon}_{ijkl}$, where ${Y}_{ijkl}$ is the observed value of genotype *l* in row *i*, column *j*, block *k*, and year *m*; *μ* is the overall mean; ${L}_{i}$ and ${C}_{j}$ are the fixed effects of row and column, respectively; ${B}_{k}$ is the fixed effect of block within year; and $\:{G}_{l}$ is the genotype effect, decomposed into ${T}_{l}^{{\prime}}$ (fixed effect of the common treatments) and ${T}_{lm}$ (random effect of the regular treatments within blocks). The genotype × year interaction ${\left(GA\right)}_{lm}$ was treated as a random effect following $N(0,{\sigma}_{GA}^{2})$, and the residual error was assumed to follow ${\epsilon}_{ijkl}\sim N(0,{\sigma}^{2})$. Separate models were fitted for each herbicide and analytical scenario; therefore, heterogeneous residual variances across years or herbicides were not modeled, and homogeneous residual variance was assumed within each fitted model. No additional weighting of de-regressed BLUPs was applied in the association analyses.

De-regressed BLUPs were calculated following Garrick et al. (2009): $BLU{P}_{dreg}=BLUP\left(1-\frac{PEV}{{\sigma}_{g}^{2}}\right)$, where PEV represents the prediction error variance ($PEV=\mathrm{V}\mathrm{a}\mathrm{r}(u-\widehat{u})$) and ${\sigma}_{g}^{2}$ is the estimated genotypic variance. The PEV associated with each genotype BLUP was obtained from the conditional posterior variance of the random genetic effects estimated using the ranef() function with the condVar = TRUE option.

Linkage disequilibrium (LD) between pairs of SNPs was calculated in R v4.4.2 (R Core Team 2025), using the genomicMateSelectR package (Ramadeu 2023). The LD.decay() function was employed to estimate the average decay of *r*^2^ as a function of physical distance between markers, an essential parameter for determining the mapping resolution of GWAS.

Population structure was assessed by principal component analysis (PCA) using the SNP marker matrix. The first five principal components were included as covariates (Q matrix) in all GWAS models to account for population structure and reduce false-positive associations (Price et al. 2006). Because the same parameterization was used throughout the study, no formal comparison among alternative numbers of principal components was performed.

Association analyses were conducted using several regression models implemented in the GAPIT v3.0 package (Wang and Zhang 2021) within the R environment (R Core Team 2025). All models simultaneously incorporated the kinship matrix (K), estimated using the VanRaden (2008) method, and the population structure covariates (Q) derived from PCA to account for relatedness among genotypes and underlying population structure.

Three modeling approaches were applied: (1) MLM (mixed linear model) – a single-locus model that accounts for fixed effects of population structure (Q) and random effects of kinship (K) (Tan and Zhao 2023); (2) MLMM (multi-locus mixed model) – an extension of MLM that incorporates multiple significant SNPs as cofactors (pseudo-QTNs), thereby increasing detection power (Zhang et al. 2023); and (3) BLINK (Bayesian information and linkage-disequilibrium iteratively nested keyway)—a model that uses Bayesian information criteria (BIC) and linkage disequilibrium patterns to iteratively select informative pseudo-QTNs, reducing computational time and minimizing model overfitting relative to previous approaches (Enyew et al. 2022).

Genome-wide significance was determined using the Bonferroni correction, with the significance threshold defined as *α=0.05/m*, where *m* is the total number of SNPs retained after quality control. Association results were visualized using Manhattan and quantile–quantile (Q–Q) plots, and significant loci were further examined according to their genomic positions and nearby candidate genes.

Genomic inflation factors (${\lambda}_{GC}$) were calculated for each GWAS analysis using the genomic control method (Devlin and Roeder 1999). P-values from the association tests were converted to chi-square statistics (1 degree of freedom), and ${\lambda}_{GC}$was estimated as the ratio between the observed median chi-square statistic and the expected median under the null hypothesis. These values were used as an additional measure of GWAS model calibration.

Significant SNPs were subsequently classified according to their consistency across GWAS models within each dataset. Markers detected by more than one model were classified as multiple-model support (MM), whereas those detected by only one model were classified as single-model evidence (SE).

### Functional annotation of candidate genes

SNPs significantly associated with herbicide tolerance were mapped against the version 7.1 reference genome of *Manihot esculenta* available in Phytozome.

(https://phytozome-next.jgi.doe.gov/). Candidate genes were identified based on physical proximity to lead SNPs using a fixed interval of ± 120 kb upstream and downstream of each significant marker.

This interval was defined a priori as a conservative locus-centered window for candidate gene identification, consistent with gene annotation approaches commonly used in genome-wide association studies based on genotyping-by-sequencing (GBS) data, where marker density and uneven genomic coverage limit high-resolution fine mapping.

Although genome-wide linkage disequilibrium (LD) in the cassava panel extends to approximately 4–6 Mb, this estimate reflects long-range background LD resulting from population structure and historical recombination rather than local mapping resolution. Consequently, genome-wide LD was not used to define candidate gene boundaries. Instead, the fixed ± 120 kb interval was adopted as a pragmatic approach to prioritize genes located near significant SNPs while accounting for variation in local LD patterns and the limited resolution of GBS-derived marker datasets. Genes overlapping or located within this interval were identified using the cassava genome annotation (GFF3) file.

Candidate genes were functionally annotated using Gene Ontology (GO) terms (Ashburner et al. 2000), which were further refined through queries to the QuickGO database (Binns et al. 2009). Genes were classified according to their associated biological processes, molecular functions, and cellular components following the Gene Ontology framework.

## Results

Based on a previous evaluation of the temporal dynamics of herbicide injury across multiple assessment dates, phytotoxicity measured at 9 days after application (PhytoX9DAA) was identified as the most informative phenotype for genetic analyses because it provided the greatest discrimination among cassava genotypes (Santiago et al. 2026). Accordingly, PhytoX9DAA was selected for all subsequent genome-wide association analyses. Although the area under the phytotoxicity progress curve (AUPPC) and the percentage reduction of accumulated phytotoxic symptoms over time (PctReduction) were also evaluated, no significant marker–trait associations were detected for either trait in any of the GWAS models (Figures S1 and S2). Therefore, only PhytoX9DAA was retained for further analyses.

Variance component analysis and likelihood ratio tests revealed high genetic contribution to phytotoxicity at 9 DAA across all herbicides evaluated (Table 1). The random effect of genotype was significant (*p* ≤ 0.001) for mesotrione, S-metolachlor, and chloransulam-methyl, indicating substantial genetic variation among cassava clones and highlighting strong potential for selection of herbicide tolerance. The genotype × year interaction was also significant for all herbicides, although of lower magnitude compared to the main genotypic effect. This indicates that while environmental conditions influenced genotype performance, the relative ranking of clones remained sufficiently consistent across years to support effective selection. Nonetheless, the presence of this interaction underscores the importance of multi-environment evaluation to identify genotypes with stable tolerance.

Among fixed effects, a significant check effect was observed only for S-metolachlor, suggesting differential responses among reference genotypes for this herbicide. Spatial variation within the experimental field was generally well controlled, although a significant row effect was detected for mesotrione, indicating localized environmental heterogeneity that was appropriately accounted for in the model. Residual variance differed among herbicides, with higher values observed for S-metolachlor relative to mesotrione and chloransulam-methyl, suggesting greater environmental sensitivity or unexplained variation for this compound.

**Table 1:** Significance tests of fixed and random effects for phytotoxicity measured at 9 days after herbicide application (PhytoX9DAA) in 194 cassava genotypes evaluated across two growing seasons (2023 and 2024)

Effect	Mesotrione	S-metolachlor	Chloransulam-methyl
Check^F^	0.22	5.04***	0.64
Row^F^	17.51***	1.09	0.05
Column^F^	0.04	0.60	1.92
Block^F^	0.69	2.19**	1.31
Genotype^R^	532.12***	391.00***	713.52***
Genotype × Year^R^	35.05***	7.63**	11.74***
σ^2^	6.80	10.12	5.14

F, fixed effect; R, random effect. *, **, and *** indicate significance at the 5, 1, and 0.1% probability levels, respectively, based on the F test (fixed effects) or likelihood ratio test (LRT; random effects). DAA, days after herbicide application; σ^2^, residual variance

### SNP marker distribution and population structure

**Fig. 1:**
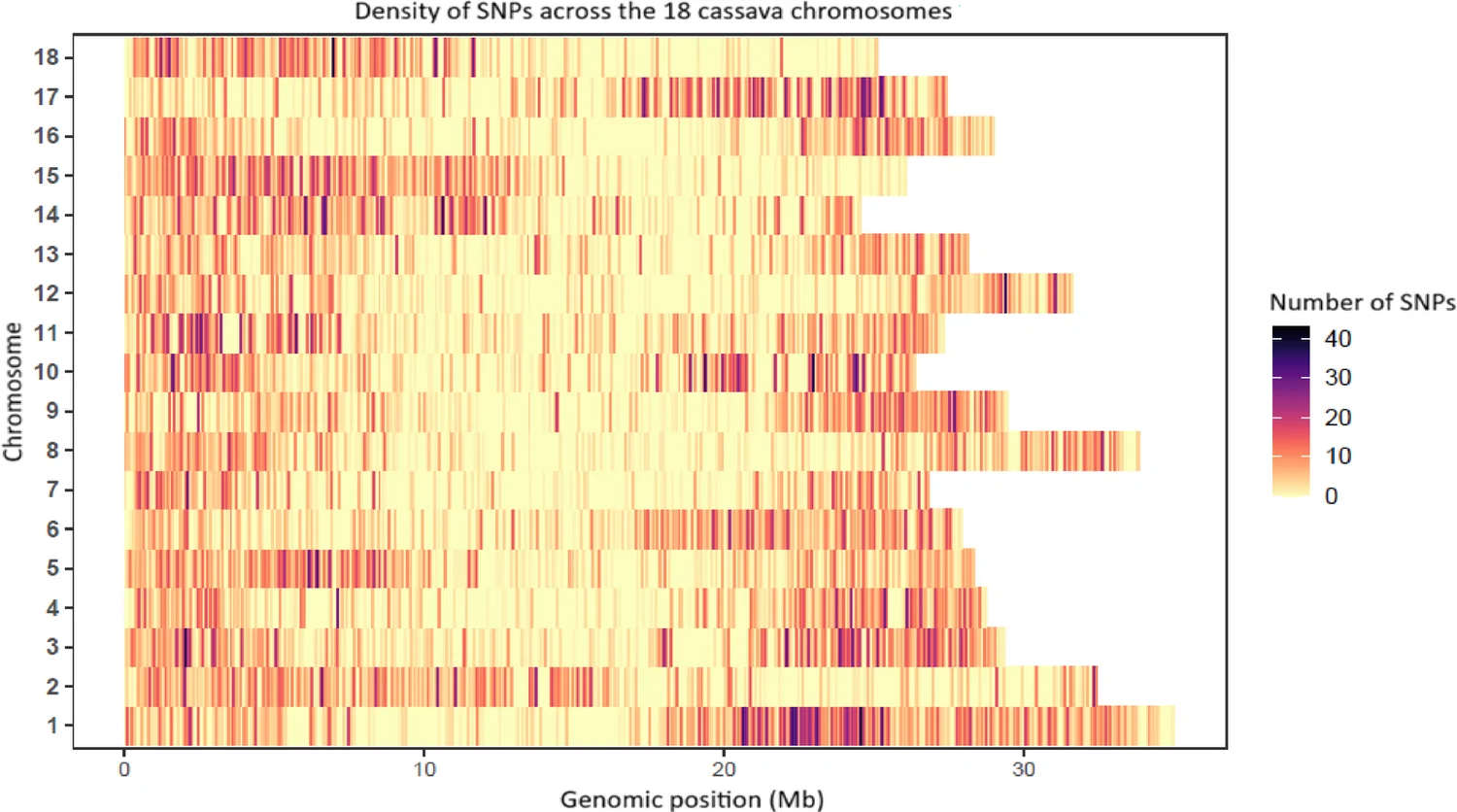
Genomic distribution and density of 23,047 single nucleotide polymorphism (SNP) markers across the 18 cassava chromosomes. Each bar represents a chromosome showing the physical positions of SNPs (in Mb), while the color scale on the right indicates the local marker density

A total of 23,047 SNPs were mapped across the 18 chromosomes of the cassava genome, covering a physical length of approximately 532.5 Mb, which corresponds to an average density of ~ 0.04 variants per kbp (Fig. 1). At the chromosome level, SNP coverage ranged from 862 markers on chromosome 7–2115 markers on chromosome 1 (Table S2). The mean minor allele frequency (MAF) was 0.169, and approximately 70% of the SNPs exhibited MAF > 5%, indicating an enrichment of common alleles within the population (Figure S3).

In addition to allele frequency, genetic diversity parameters were estimated to characterize the variability of the panel. The expected heterozygosity (*H*_e_) showed an average value of 0.240, while the observed heterozygosity (*H*_o_) was 0.235. The close agreement between *H*_e_ and *H*_o_ indicates the absence of pronounced deviations in genotype frequencies and suggests the maintenance of moderate genetic diversity in the evaluated panel. The distribution of heterozygosity values among the genotypes is consistent with the patterns observed for minor allele frequency (MAF) and the rapid decay of linkage disequilibrium. These results confirm that the analyzed population presents sufficient genetic diversity to support genome-wide association analyses with adequate resolution.

**Fig. 2:**
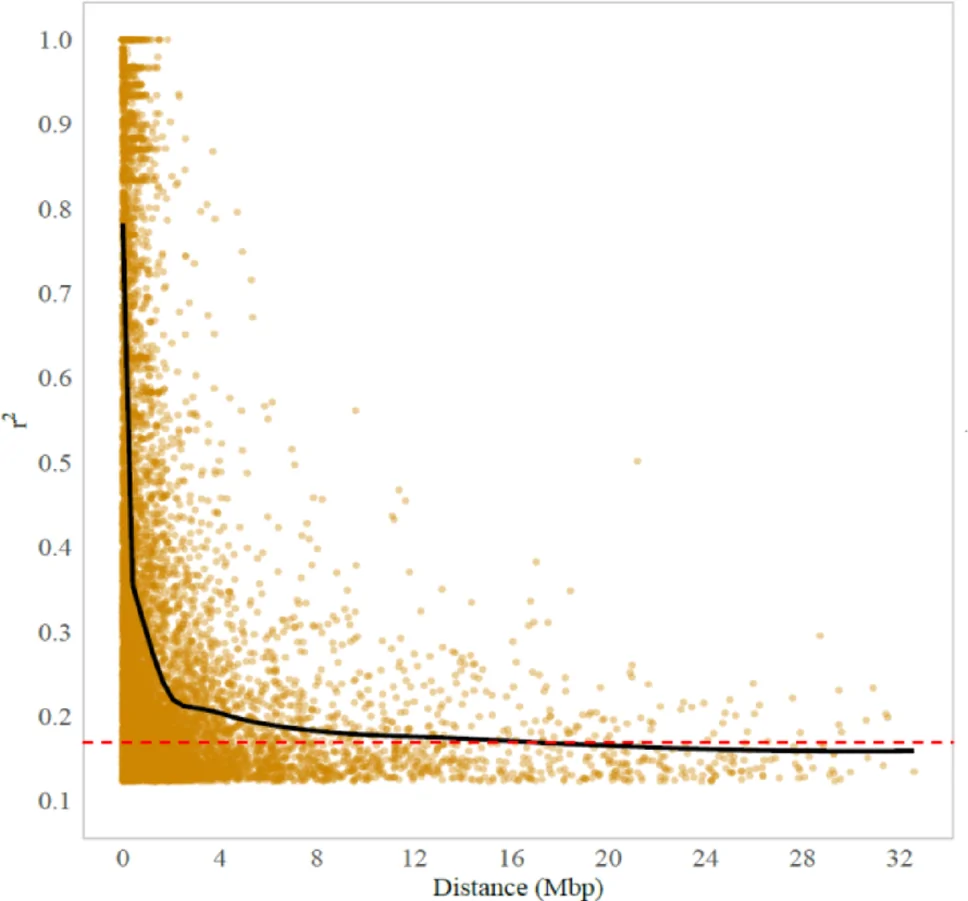
Linkage disequilibrium (LD) decay as a function of genomic physical distance between pairs of single nucleotide polymorphism (SNP) markers in the cassava genotype panel. Each orange point represents the squared correlation coefficient (*r*^2^) between two markers. The solid black line represents the average LD decay trend, whereas the red dashed line indicates the background LD level (*r*^2^ = 0.16) estimated from SNP pairs separated by more than 10 Mbp

Genome-wide linkage disequilibrium (LD) analysis revealed a rapid decline in *r*^2^ values as the physical distance between markers increased (Fig. 2). The background LD level, estimated using SNP pairs separated by more than 10 Mbp, was *r*^2^ = 0.16. The observed pattern indicates that LD in the evaluated cassava population decays rapidly, reaching values close to the background level at approximately 4–6 Mbp. This pattern reflects the high genetic diversity and recombination frequency within the panel, providing adequate resolution for genome-wide association analyses (GWAS).

**Fig. 3:**
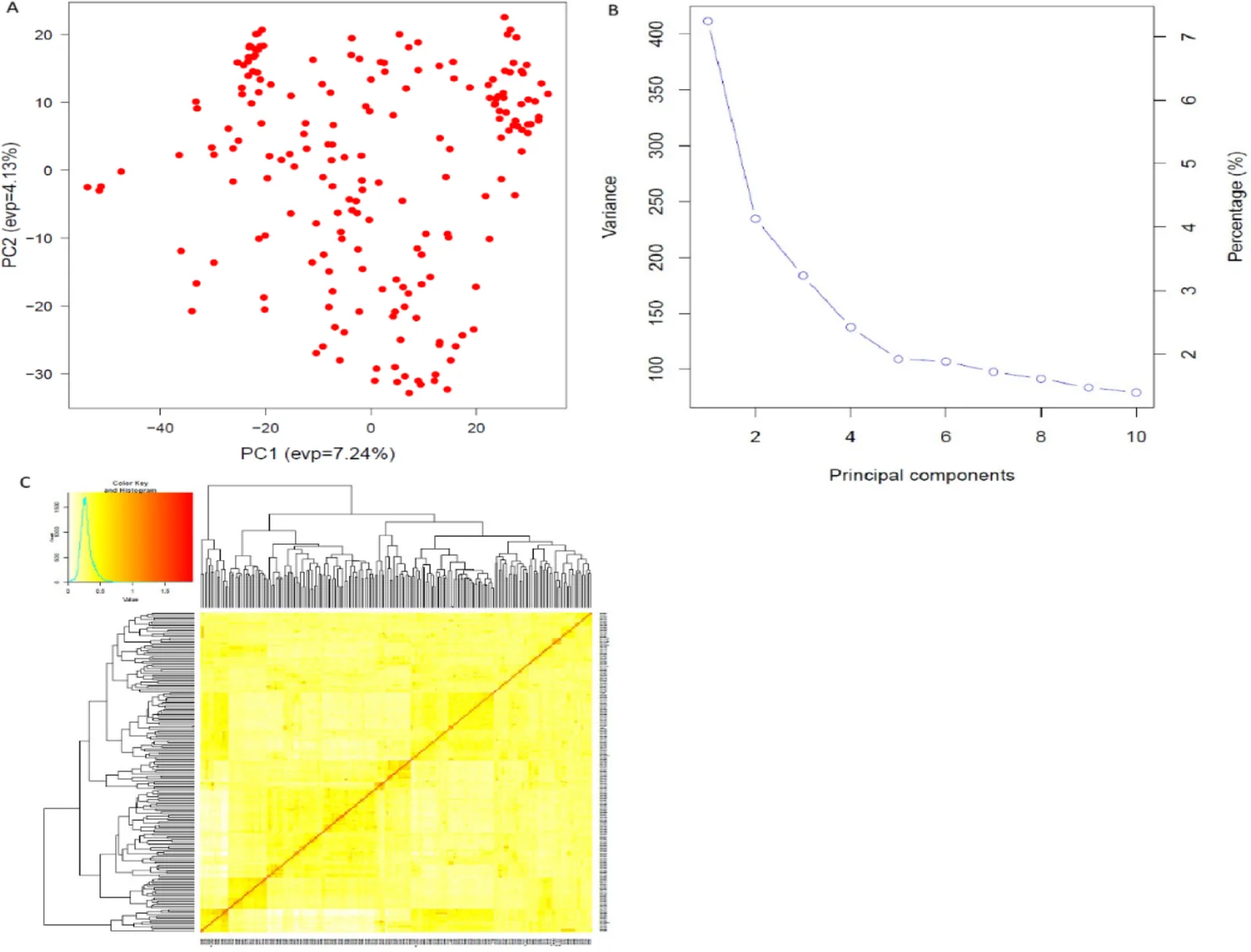
Population structure and genetic relatedness among 194 cassava genotypes based on 23,047 SNP markers. Principal component analysis (PCA) was performed using the SNP genotype matrix to investigate the population structure of the panel. **A** Scatter plot of genotypes along the first two principal components (PC1 = 7.24% and PC2 = 4.13%). **B** Percentage of variance explained by the first ten principal components. **C** Heatmap of the kinship matrix estimated based on identity-by-state (IBS)

Principal component analysis (PCA) was performed to assess the population structure among the 194 cassava genotypes. The first two principal components (PC1 and PC2) explained 7.24 and 4.13% of the total genetic variance, respectively, corresponding to 11.37% of the combined variance (Fig. 3A). Components PC3 through PC10 explained progressively smaller proportions of the total variance, indicating that only a limited fraction of the genetic variation is attributable to population structure (Fig. 3B).

The identity-by-state (IBS) distance matrix revealed substantial variation in the degree of relatedness within the panel, with a mean value of 0.287, ranging from 0 to 1.091. Clusters of closely related individuals were observed along the diagonal of the matrix, whereas regions outside the diagonal indicated low levels of relatedness among genotypes (Fig. 3C).

Genomic inflation factors (λGC) were calculated as an additional measure of GWAS model calibration (Table S3). Across all analyses, λGC values ranged from 0.62 to 1.07. BLINK and MLMM produced λGC values close to 1.0, indicating appropriate calibration, whereas MLM yielded λGC values below 1.0, consistent with conservative test statistics. Together with the Q–Q plots, these results indicate that the GWAS models were adequately calibrated and support the reliability of the detected marker–trait associations.

### GWAS results

To facilitate the interpretation of GWAS results across the different analytical approaches, significant SNPs were classified according to the consistency of their detection among GWAS models. Markers identified by more than one model within the same dataset were classified as having multiple-model support (MM), whereas markers detected by only one model were classified as single-model evidence (SE). This classification summarizes the overlap among BLINK, MLM, and MLMM while retaining all statistically significant model-specific associations (Table S4).

Statistical significance was determined independently for each GWAS model using the Bonferroni-adjusted genome-wide significance threshold (α = 0.05). The MM/SE classification was subsequently applied as a post hoc measure of consistency across analytical frameworks rather than as an alternative significance criterion. Accordingly, MM markers represent loci consistently detected by multiple GWAS models, whereas SE markers represent model-specific associations that may reflect differences in model sensitivity, allele frequency, or statistical power and should therefore be interpreted with greater caution.

#### Chloransulam-methyl

No SNPs significantly associated with tolerance to chloransulam-methyl were detected in any GWAS model or analytical scenario. The absence of significant associations may reflect a complex genetic architecture involving numerous loci of small effect but may also result from limited statistical power, insufficient marker density, low-frequency causal variants, or characteristics of the phenotypic assessment that reduced the ability to detect significant marker–trait associations.

#### S-metolachlor

For S-metolachlor, a single significant SNP (chr9:1858429) located on chromosome 9 was detected exclusively by the BLINK model. This marker was consistently detected across all analytical scenarios (2023, 2024, and the combined analysis) by the BLINK model, with *p*-values ranging from 1.50 × 10^−6^ to 1.67 × 10^−6^ (Table 2). The SNP had a minor allele frequency (MAF) of 0.023, indicating that the associated allele is rare within the evaluated panel. Although this marker was consistently detected by the BLINK model across both individual years and the combined analysis, it was not supported by the MLM or MLMM models. Consequently, despite the reproducibility of this statistical association within the BLINK framework, its low allele frequency and lack of support from alternative GWAS models indicate that the biological relevance of this locus should be interpreted cautiously until independent validation and estimates of allele effects become available.

**Fig. 4:**
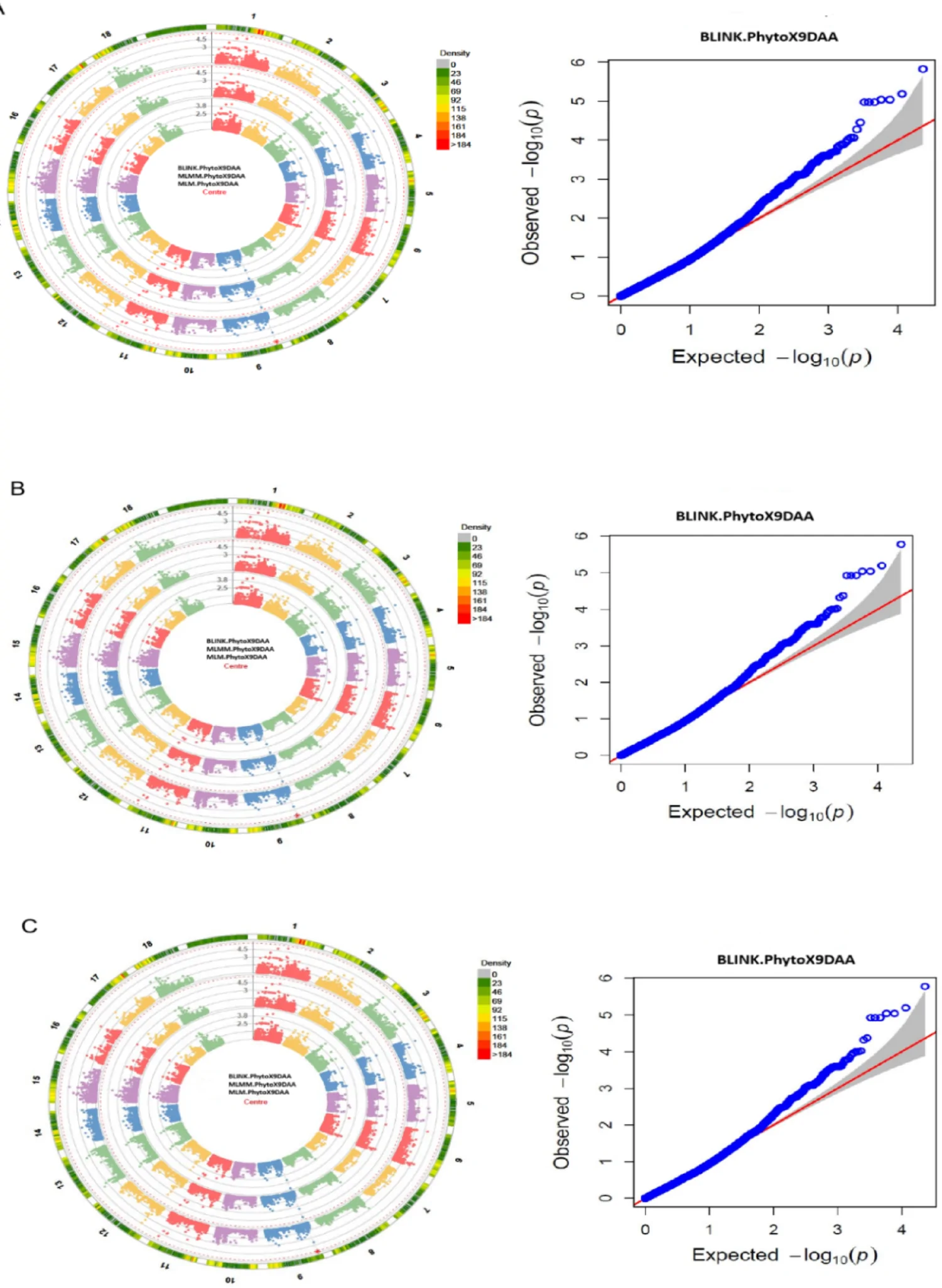
Genome-wide association study (GWAS) results for phytotoxicity assessed 9 days after herbicide application (9 DAA) following S-metolachlor treatment in 2023 (**A**), 2024 (**B**), and in the combined analysis (**C**) of a panel of 194 cassava genotypes. Left panel: circular Manhattan plots showing − log_10_(*p*) values across genomic positions. Right panel: Q–Q plots for the BLINK model (Bayesian information and linkage disequilibrium iteratively nested keyway)

Quantile–quantile (Q–Q) plots showed good agreement with the expected null distribution. However, a slight upward deviation from the expected line was observed prior to the highly significant tail, beginning around expected − log_10_(*p*) ≈ 1.5–2.0, which is consistent with the presence of a true association signal (Fig. 4). No additional SNPs exceeded the genome-wide significance threshold, suggesting that only a limited number of loci associated with phytotoxic response to S-metolachlor were detectable with the current marker density, whereas additional loci with smaller effects may have remained below the detection threshold.

**Table 2:** Significant SNPs (Bonferroni correction, α = 0.05) associated with tolerance to the herbicide S-metolachlor in cassava genotypes evaluated in 2023, 2024, and in the combined analysis (2023 + 2024)

Model	SNP	Chromosome	Position (bp)	MAF	Year	*P*-value
BLINK	chr9:1858429	9	1,858,429	0.023	2023	1.50E^−06^
					2024	1.67E^−06^
					Combined	1.67E^−06^

SNPs were identified using the MLM (mixed linear model), MLMM (multi-locus mixed model), and BLINK (Bayesian information and linkage-disequilibrium iteratively nested keyway) models for phytotoxicity assessed 9 days after herbicide application (PhytoX9DAA)

MAF, minor allele frequency

#### Mesotrione

A total of 68 significant SNP associations with PhytoX9DAA were identified for mesotrione, distributed across multiple chromosomes and detected by the BLINK, MLM, and MLMM models (Table 3). These findings indicate a complex genetic architecture underlying mesotrione tolerance, involving multiple loci contributing to the observed phenotype.

**Fig. 5:**
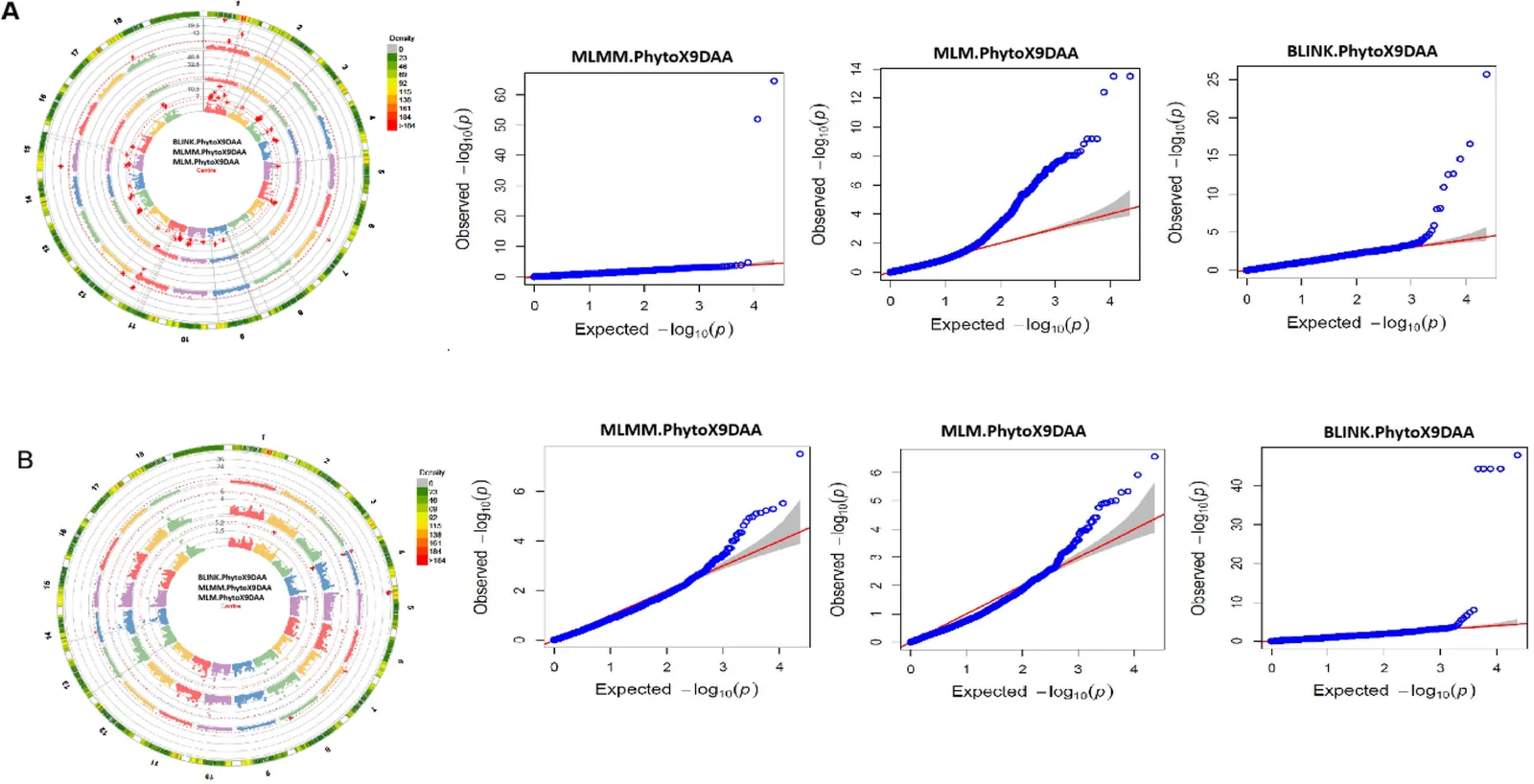
Genome-wide association study (GWAS) results for phytotoxicity (PhytoX9DAA) assessed 9 days after mesotrione application, evaluated in 2024 (**A**) and in the combined analysis (**B**) across 194 cassava genotypes. Left panel: circular Manhattan plots displaying − log_10_(*p*) values across genomic positions. Right panel: Q–Q plots for the MLMM (multi-locus mixed model), MLM (mixed linear model), and BLINK (Bayesian information and linkage disequilibrium iteratively nested keyway) models, illustrating model fit and genomic inflation

In the 2024 analysis, BLINK identified eight significant SNPs primarily located on chromosomes 1, 7, 11, 15, and 18, with p-values as low as 2.27 × 10^−26^. The MLM model detected 55 significant SNPs across most chromosomes (p-values ranging from 3.18 × 10^−14^ to 8.28 × 10^−8^), whereas MLMM identified only two highly significant SNPs on chromosome 11, with a minimum *p*-value of 2.45 × 10^−65^. The strongest association peaks were observed on chromosomes 1 (chr1:11468003, chr1:24877106, and chr1:16899387), 7 (chr7:781893), 11 (chr11:25011154 and chr11:25007496), 15 (chr15:3257801), and 18 (chr18:8669973), as identified by BLINK and supported by MLM and MLMM (Fig. 5).

In the combined analysis, BLINK identified 11 significant SNPs located on chromosomes 4, 5, 7, and 14, with p-values ranging from 1.57 × 10^−48^ to 2.66 × 10^−7^. Notably, four adjacent SNPs on chromosome 5 (chr5:4263587, chr5:4263683, chr5:4321487, and chr5:4431525) indicate a local linkage disequilibrium (LD) block, likely representing a haplotype associated with mesotrione tolerance.

The MLM model identified two additional SNPs on chromosomes 2 and 4 (*p* = 1.21 × 10^−6^ and 2.73 × 10^−7^), while MLMM confirmed a highly significant SNP on chromosome 4 (chr4:1751307; *p* = 3.08 × 10^−8^). Additional loci reinforced signals detected in the individual-year analyses. In the combined dataset, the most relevant markers were located on chromosomes 5 (chr5:3896743, chr5:4263587, chr5:4321487, and chr5:4431525), 4 (chr4:1751307), 7 (chr7:1714750), and 14 (chr14:8736846). Chromosome 5 harbored the strongest signals, with *p*-values ranging from 1.57 × 10^−48^ to 5.18 × 10^−45^. The association at chr4:1751307 was consistently supported by both MLM and MLMM (Fig. 5).

**Table 3:** SNP (Single Nucleotide Polymorphisms) markers significantly associated (Bonferroni correction, α = 0.05) with tolerance to mesotrione in 194 cassava genotypes evaluated in 2023, 2024, and in the combined analysis

Year	Model	SNP	Chromosome	Position (bp)	*P*-value	MAF
2024	BLINK	chr1:11468003	1	11,468,003	2.27E-26	0.010
		chr1:24877106	1	24,877,106	2.96E-17	0.012
		chr7:781893	7	781,893	2.93E-15	0.010
		chr15:3257801	15	3,257,801	2.50E-13	0.015
		chr11:25011154	11	25,011,154	3.16E-13	0.021
		chr11:25007496	11	25,007,496	1.41E-11	0.018
		chr18:8669973	18	8,669,973	7.89E-09	0.106
		chr1:16899387	1	16,899,387	1.01E-08	0.023
2024	MLM	chr11:25011154	11	25,011,154	3.18E-14	0.021
		chr11:25011189	11	25,011,189	3.18E-14	0.021
		chr1:16899387	1	16,899,387	3.94E-13	0.021
		chr1:11468003	1	11,468,003	6.40E-10	0.008
		chr1:11468040	1	11,468,040	6.40E-10	0.008
		chr1:11468043	1	11,468,043	6.40E-10	0.008
		chr1:24877106	1	24,877,106	1.49E-09	0.013
		chr3:23977150	3	23,977,150	4.76E-09	0.010
		chr3:2168588	3	2,168,588	5.75E-09	0.010
		chr18:9235000	18	9,235,000	9.35E-09	0.039
		chr18:9235026	18	9,235,026	9.35E-09	0.039
		chr6:25096573	6	25,096,573	9.71E-09	0.010
		chr6:25096588	6	25,096,588	9.71E-09	0.010
		chr6:25096622	6	25,096,622	9.71E-09	0.010
		chr13:24594495	13	24,594,495	1.32E-08	0.010
		chr4:5900479	4	5,900,479	1.87E-08	0.039
		chr10:20202043	10	20,202,043	1.92E-08	0.036
		chr6:26611303	6	26,611,303	2.10E-08	0.039
		chr9:21874919	9	21,874,919	2.17E-08	0.010
		chr10:22980602	10	22,980,602	2.17E-08	0.010
		chr1:7460013	1	7,460,013	2.70E-08	0.013
		chr1:7460086	1	7,460,086	2.70E-08	0.010
		chr13:2462717	13	2,462,717	3.78E-08	0.010
		chr13:2462732	13	2,462,732	3.78E-08	0.010
		chr15:21723327	15	21,723,327	4.03E-08	0.013
		chr4:3015128	4	3,015,128	4.86E-08	0.028
		chr3:2920362	3	2,920,362	6.57E-08	0.057
		chr15:6464755	15	6,464,755	8.28E-08	0.010
		chr15:6687927	15	6,687,927	8.28E-08	0.010
		chr15:6729375	15	6,729,375	8.28E-08	0.010
		chr15:6729388	15	6,729,388	8.28E-08	0.010
2024	MLMM	chr11:25011154	11	25,011,154	2.45E-65	0.021
		chr11:25007496	11	25,007,496	9.61E-53	0.018
Combined	BLINK	chr5:3896743	5	3,896,743	1.57E-48	0.026
		chr5:4263587	5	4,263,587	5.18E-45	0.023
		chr5:4263683	5	4,263,683	5.18E-45	0.023
		chr5:4321487	5	4,321,487	5.18E-45	0.023
		chr5:4431525	5	4,431,525	5.18E-45	0.023
		chr7:1714750	7	1,714,750	1.02E-08	0.031
		chr4:1751307	4	1,751,307	4.48E-08	0.018
		chr14:8736846	14	8,736,846	2.66E-07	0.018
Combined	MLM	chr4:1751307	4	1,751,307	2.73E-07	0.018
		chr2:12804185	2	12,804,185	1.21E-06	0.072
Combined	MLMM	chr4:1751307	4	1,751,307	3.08E-08	0.018

Associations were identified using the MLM (mixed linear model), MLMM (multi-locus mixed model), and BLINK (Bayesian information and linkage-disequilibrium iteratively nested keyway) models, based on phytotoxicity (PhytoX) assessed nine days after herbicide application (9 DAA)

MAF, minor allele frequency

### Candidate gene annotation

Significant SNPs associated with herbicide tolerance, identified using the BLINK, MLM, and MLMM models, were used to identify candidate genes based on the cassava reference genome (*Manihot esculenta* Crantz, v7.1; Phytozome v12.1). For each SNP, a 240 kb genomic window (± 120 kb) was defined to capture genes potentially in linkage disequilibrium with the associated loci. This approach enabled the identification of genomic regions likely underlying the observed tolerance responses.

A total of 59 candidate genes were identified across genomic regions associated with herbicide response (Table S5). Functional annotation based on Gene Ontology (GO) terms and protein descriptions indicated genes putatively involved in membrane transport, channel activity, protein phosphorylation, signal transduction, transcriptional regulation, RNA processing, and metabolic regulation.

To facilitate locus-by-locus interpretation of the strongest GWAS signals, Table 4 summarizes the lead SNPs, candidate genes identified within the predefined ± 120 kb interval, simplified Gene Ontology (GO) annotations, and supporting evidence from orthologous genes or detoxification pathways reported in other plant species. This summary provides a more direct and traceable link between the statistical associations and their biological interpretation while emphasizing that the proposed functions are based on gene annotation and comparative evidence rather than experimental validation in cassava.

On chromosome 1, the genes *Manes.01G088600*, *Manes.01G088700*, and *Manes.01G088800* were associated with RNA binding, RNA processing, and RNA methyltransferase activity, suggesting a role for post-transcriptional regulation in herbicide response.

On chromosome 7, the genes *Manes.07G005800*, *Manes.07G005900*, and *Manes.07G006000* were annotated with antiporter activity, membrane localization, and xenobiotic transmembrane transporter activity. These functions are typically associated with transporter proteins from the ABC (ATP-binding cassette) and MATE (Multidrug and Toxic Compound Extrusion) families, which play key roles in xenobiotic sequestration and efflux across plant species.

On chromosome 15, *Manes.15G039600*, *Manes.15G039700*, and *Manes.15G039800* were annotated with DNA binding, DNA-binding transcription factor activity, regulation of DNA-templated transcription, and transmembrane transport. Because these genes are located near genomic regions showing strong association signals, they represent plausible candidates for involvement in transcriptional regulation and cellular transport processes associated with herbicide response.

Additional candidate genes identified on chromosomes 2, 9, and 15 were annotated with functions related to membrane transport, signal transduction, and metabolic regulation. In particular, *Manes.02G151900* and *Manes.02G152700* were associated with channel activity, transmembrane transport, and signal peptide processing, whereas *Manes.09G060900* was annotated with protein kinase activity, ATP binding, and protein phosphorylation. On chromosome 15, *Manes.15G083800* and *Manes.15G084000* were annotated with S-adenosylmethionine-dependent methyltransferase activity, protein kinase activity, ATP binding, protein phosphorylation, and membrane-associated functions. These annotations suggest that cellular signaling, membrane transport, and metabolic regulation may contribute to cassava responses to herbicide exposure. However, these functional interpretations are based on annotation and comparative evidence and should be considered hypotheses requiring experimental validation.

**Table 4:** Summary of the lead GWAS loci associated with herbicide tolerance in cassava (*Manihot esculenta* Crantz), including lead SNPs, nearby candidate genes, simplified Gene Ontology (GO) annotations, and supporting functional evidence from orthologous genes reported in other plant species

Lead SNP	Chr	Position (bp)	Herbicide	Candidate gene(s)	Function (GO simplified)	Supporting evidence
chr11:25011154	11	25,011,154	Mesotrione	Manes.11G107000; Manes.11G107050; Manes.11G107101	Oxidoreductase/monooxygenase	P450s involved in mesotrione detoxification in rice (Xu et al. 2024).
chr11:25007496	11	25,007,496	Mesotrione	Manes.11G106900; Manes.11G107000; Manes.11G107050	Oxidoreductase/acyltransferase	P450-mediated metabolism linked to mesotrione tolerance in rice (Xu et al. 2024).
chr1:24877106	1	24,877,106	Mesotrione	Manes.01G088600; Manes.01G088700; Manes.01G088800	RNA processing/binding	No functional evidence reported.
chr1:11468003	1	11,468,003	Mesotrione	No annotated gene identified	–	–
chr4:1751307	4	1,751,307	Mesotrione	Manes.04G013000; Manes.04G013101	Metal ion binding	No functional evidence reported.
chr9:1858429	9	1,858,429	S-metolachlor	Manes.09G008600; Manes.09G008801; Manes.09G009000	DNA binding/methyltransferase	GST/P450/ABC-MATE detoxification pathways reported in other plant species; no direct evidence for the candidate genes (Cummins et al. 2013; Xia et al. 2021; Goldberg-Cavalleri et al. 2023; Xu et al. 2024; Rigon et al. 2025).

Candidate genes were identified within a predefined ± 120 kb interval surrounding each lead SNP. Functional interpretations are based on gene annotation and comparative evidence and do not represent experimental validation in cassava

## Discussion

An important methodological consideration for interpreting the present results is the selection of the phenotype used for the GWAS analyses. Although chloransulam-methyl generally induces visible injury more slowly than mesotrione and S-metolachlor, post-emergence symptoms are typically first observed between 3 and 10 days after application (DAA) (Shaner 2014). Consistent with this pattern, segmented regression analyses indicated that the period between 9 and 11 DAA corresponded to the maximum expression of phytotoxicity and the greatest phenotypic differentiation among cassava genotypes (Santiago 2026). In addition, variance component analysis revealed a highly significant genotypic effect for phytotoxicity evaluated at 9 DAA across all herbicides (Table 1), demonstrating substantial genetic variation for this trait. These findings support the use of phytotoxicity at 9 DAA as the phenotype for GWAS because this time point captured the greatest genetic differentiation among cassava genotypes while providing a common evaluation stage for all herbicides. Although symptom development continued beyond this period for the ALS inhibitor chloransulam-methyl, the 9 DAA assessment provided sufficient phenotypic differentiation for genome-wide association analyses.

To the best of our knowledge, this study is among the first GWAS investigations specifically examining cassava tolerance to mesotrione, S-metolachlor, and chloransulam-methyl by integrating three complementary GWAS models with phenotypic evaluations conducted across two growing seasons. Our results revealed no significant SNPs associated with tolerance to chloransulam-methyl, a single consistent SNP associated with S-metolachlor (chr9:1858429; MAF = 0.023), and multiple association peaks for mesotrione distributed across several chromosomes. These findings confirm the presence of genetic variability in cassava responses to herbicides and highlight a heterogeneous genetic architecture across the compounds evaluated. Candidate genes identified near the associated loci were annotated with functions related to membrane transport, channel activity, protein phosphorylation, signal transduction, transcriptional regulation, RNA processing, and metabolic regulation. Collectively, these annotations provide plausible hypotheses regarding biological processes that may contribute to herbicide responses in cassava; however, these functional interpretations are based on gene annotation and comparative evidence rather than direct experimental validation.

The significant genotype × year interaction observed for all herbicides indicates that environmental conditions contributed to variation in herbicide responses among cassava genotypes. This finding is consistent with the well-established influence of environmental conditions on herbicide absorption, translocation, metabolism, and phytotoxicity. Despite the significant genotype × year interaction, the chromosome 9 SNP associated with S-metolachlor (chr9:1858429) was consistently detected by the BLINK model in both years and in the combined analysis, indicating a reproducible statistical association under the evaluated conditions. Nevertheless, because this association was supported by only one GWAS model and involved a rare allele (MAF = 0.023), its biological relevance should be interpreted cautiously until independent validation and effect-size estimates become available. In contrast, most mesotrione-associated loci were detected in only one analytical dataset, suggesting that some genomic regions may be more sensitive to environmental variation. For chloransulam-methyl, the absence of significant associations may likewise have been influenced by genotype × year interaction, in addition to limited statistical power and the possibility of a more complex genetic architecture.

### GWAS results for chloransulam-methyl

Unlike mesotrione and S-metolachlor, no significant SNPs associated with chloransulam-methyl tolerance were detected under any of the GWAS models evaluated. This absence of detectable associations should be interpreted cautiously. One possible explanation is that tolerance to this herbicide is controlled by numerous loci with individually small effects, each contributing only modestly to the phenotypic variation and therefore remaining below the genome-wide significance threshold. However, alternative explanations are equally plausible. The relatively small population size, limited marker density, low-frequency causal variants not represented in the SNP dataset, and the limited resolution of the phenotypic assessment may all have reduced the statistical power to detect significant marker–trait associations. Consequently, the present results do not demonstrate a polygenic genetic architecture but are consistent with this possibility. Additional studies involving larger populations, higher-density genotyping, and complementary mapping approaches will be required to clarify the genetic basis of chloransulam-methyl tolerance.

### Genomic associations and variability for S-metolachlor

For S-metolachlor, GWAS identified a single significant SNP (chr9:1858429), detected exclusively by the BLINK model in 2023, 2024, and the combined analysis. The recurrence of this association across both growing seasons and the combined dataset indicates a reproducible statistical signal under the evaluated conditions. However, the associated allele occurred at low frequency (MAF = 0.023), and the association was not supported by the MLM or MLMM models. Therefore, its biological relevance should be interpreted cautiously until confirmed through independent populations, allele-effect estimation, and functional validation.

Similar genetic patterns have been reported in other crop species. In soybean, Jo et al. (2023) identified a limited number of SNPs associated with tolerance to bentazon, clustered on chromosome 5 and harboring genes encoding ABC (ATP-binding cassette) transporters and ankyrin repeat proteins (ANK), both involved in xenobiotic transport and intracellular compartmentalization. Similarly, Abou-Khater et al. (2022) reported loci associated with tolerance to metribuzin and imazethapyr in faba bean (*Vicia faba* L.), including genes encoding protein kinases and acidic endochitinases, which are implicated in antioxidant responses and cellular detoxification.

S-metolachlor belongs to the chloroacetanilide class of herbicides and acts by inhibiting the biosynthesis of very-long-chain fatty acids (VLCFAs), thereby disrupting lipid elongation and impairing meristematic growth. Natural or acquired tolerance to this herbicide has been widely associated with detoxification pathways, particularly those mediated by glutathione S-transferases (GSTs), which catalyze the conjugation of herbicides with glutathione, facilitating their detoxification and subsequent compartmentalization (Cummins et al. 2013; Xu et al. 2024). In addition, cytochrome P450 monooxygenases (CYPs) and membrane transporters from the ABC and MATE families play key roles in the oxidation, transport, and sequestration of xenobiotics (Xia et al. 2021; Goldberg-Cavalleri et al. 2023; Rigon et al. 2025).

The chromosome 9 region identified in this study contains candidate genes involved in regulatory processes that may influence herbicide responses. Gene annotations in this region include functions related to DNA binding, DNA replication, and methyltransferase activity. Although metabolic resistance to S-metolachlor has frequently been associated with GSTs, cytochrome P450 monooxygenases, and membrane transporters in other plant species, no direct functional evidence links the annotated cassava genes identified here to these detoxification mechanisms. Consequently, this genomic region should be regarded as a candidate locus that warrants further fine mapping and functional validation.

### Genomic associations and variability for herbicide phytotoxicity in cassava

The response to mesotrione revealed a complex genetic architecture, with multiple SNPs significantly associated with phytotoxicity distributed across chromosomes 1, 4, 5, 7, 11, 14, 15, and 18, varying across years and analytical models. The BLINK model showed higher sensitivity in detecting loci with small effects, identifying consistent association peaks at chr1:11468003, chr1:24877106, chr7:781893, and chr11:25011154, which represent key candidate regions underlying differential responses to mesotrione.

Genomic inflation factors (λGC) ranged from 0.62 to 1.07 across all GWAS analyses, indicating adequate model calibration. BLINK and MLMM produced λGC values close to 1.0, whereas MLM yielded values below 1.0, consistent with conservative test statistics rather than genomic inflation. These results agree with the corresponding Q–Q plots and support the reliability of the GWAS analyses.

The Q–Q plots showed a slight deviation from the expected null distribution in the upper tail. Such patterns are commonly observed in GWAS of complex traits and may reflect the combined effects of numerous loci with small effects together with extended linkage disequilibrium (Yu et al. 2006; Kang et al. 2010; Segura et al. 2012). Importantly, the λGC values indicate that these deviations are unlikely to result from substantial genomic inflation, supporting the overall calibration of the association models. The presence of multiple significant loci distributed across different chromosomes is therefore consistent with a quantitative genetic architecture underlying mesotrione tolerance.

Given that mesotrione tolerance is metabolically complex, involving detoxification pathways mediated by cytochrome P450 monooxygenases, glutathione S-transferases, and other oxidative enzymes, part of the observed inflation likely reflects the underlying genetic architecture of the trait (Ma et al. 2013). Similar Q–Q plot behavior has been reported in rice GWAS for herbicide resistance Xu et al. (2024), whereas studies in wheat have shown well-calibrated distributions depending on species and model choice (Kurya et al. 2022). These differences highlight the importance of interpreting Q–Q plots in light of both biological complexity and model assumptions.

Mesotrione acts by inhibiting 4-hydroxyphenylpyruvate dioxygenase (HPPD), leading to the accumulation of toxic intermediates and subsequent chlorophyll degradation. In rice, Xu et al. (2024) demonstrated that resistance to mesotrione is polygenic, involving both target-site variation (HPPD) and non-target-site resistance mechanisms, particularly those mediated by cytochrome P450 enzymes and monooxygenases. The identification of multiple loci across different cassava chromosomes is consistent with this mode of action, suggesting the involvement of detoxification pathways and phenolic metabolism in modulating herbicide response.

The combined analysis (2023 + 2024) further highlighted the importance of genomic regions on chromosomes 4, 5, and 7, particularly SNPs chr4:1751307, chr5:4263587, and chr7:1714750, which were detected by multiple models, indicating robust and consistent effects. Previous GWAS studies in cassava (Rabbi et al. 2022) have also reported associations on these chromosomes for traits related to plant architecture, productivity, and physiological performance. The recurrence of association signals in these genomic regions suggests the presence of candidate genes involved in growth regulation, metabolism, and resource allocation. Based on gene annotation and functional information available from other plant species, these biological processes may contribute to herbicide responses in cassava. However, no functional validation was performed in the present study, and these hypotheses require further investigation.

The cassava genome is characterized by moderate heterozygosity and a high proportion of repetitive sequences, which can complicate read alignment and variant calling (Amelework et al. 2021; Landi et al. 2023). Regions with high structural similarity may hinder the precise discrimination of homologous loci, potentially affecting SNP detection in short-read sequencing approaches.

In this study, stringent quality control filters were applied, including thresholds for call rate, minor allele frequency, and the incorporation of population structure and kinship matrices in the association models, standard practices to minimize spurious associations. Nonetheless, further validation through fine mapping, independent population testing, and functional characterization of candidate genes will be essential to confirm the biological relevance of the identified loci.

### Functional annotation and identification of candidate genes

Functional annotation of genes located within genomic intervals surrounding significant SNPs (Table S5) revealed functional categories associated with membrane transport, channel activity, signal peptide processing, protein phosphorylation, cellular signaling, transcriptional regulation, RNA processing, and metabolic regulation. These annotations suggest that the genomic regions associated with herbicide response contain genes potentially involved in xenobiotic transport, redox homeostasis, and metabolic detoxification, consistent with observations reported in other crop species (Adhikari et al. 2020; Abou-Khater et al. 2022; Aghogho et al. 2024). However, these putative functions are based on gene annotation and comparative evidence and have not been experimentally validated in cassava.

Although genome-wide linkage disequilibrium (LD) in the association panel extends to approximately 4–6 Mb, this estimate reflects long-range LD resulting from population structure and historical recombination rather than local mapping resolution. Consequently, genome-wide LD was not used to define candidate gene boundaries. Instead, a fixed ± 120 kb interval surrounding each lead SNP was adopted as a pragmatic, locus-centered approach to prioritize nearby candidate genes while limiting the inflation of candidate regions caused by extended LD. Candidate gene identification should therefore be regarded as proximity-based prioritization rather than an exhaustive representation of all genes potentially associated with each locus.

Several genomic regions contained genes with annotations compatible with biological processes previously implicated in herbicide responses. On chromosomes 1, 7, and 15, candidate genes including *Manes.01G088600*, *Manes.01G088700*, *Manes.01G088800*, *Manes.07G005800*, *Manes.07G005900*, *Manes.07G006000*, *Manes.15G039600*, *Manes.15G039700*, and *Manes.15G039800* were annotated with functions related to RNA processing, transcriptional regulation, DNA binding, and membrane transport. Although these genes represent plausible candidates because of their proximity to significant SNPs, their involvement in herbicide tolerance remains hypothetical and requires functional validation.

The genes *Manes.02G151900* and *Manes.02G152700*, both located on chromosome 2, are annotated with functions related to channel activity, transmembrane transport, and signal peptide processing. These features suggest potential roles in ionic flux regulation and the transport of hydrophobic molecules across cellular membranes, processes essential for maintaining cellular homeostasis and mediating the movement of metabolites and xenobiotics, although these roles remain putative in cassava. Such functions are typically associated with transporter families such as ABC (ATP-binding cassette) and MATE (multidrug and toxic compound extrusion), which are widely implicated in metabolic herbicide resistance (Goldberg-Cavalleri et al. 2023; Biswal et al. 2025).

In *Vicia faba* L., ABC and MATE transporters have been directly linked to the efflux of herbicides and their conjugated metabolites, contributing to differential tolerance among genotypes (Abou-Khater et al. 2022). Similarly, recent genomic studies in cassava have identified membrane transport genes associated with defense and physiological resistance processes (Ospina et al. 2024), suggesting conserved detoxification and homeostasis mechanisms across species.

Another important functional group includes genes such as *Manes.09G060900* and *Manes.15G083800*, associated with protein kinase activity, ATP binding, protein phosphorylation, ADP binding, and methyltransferase-related functions. These genes are annotated with functions associated with cellular signaling and metabolic regulation and may contribute to differential herbicide responses among cassava genotypes. In plants, herbicide tolerance is often associated with metabolic detoxification pathways, including reactions mediated by cytochrome P450 monooxygenases that increase the polarity of xenobiotic compounds (Yu and Powles 2014; Gaines et al. 2020). In phase II, enzymes such as glutathione S-transferases (GSTs) and UDP-glycosyltransferases (UGTs) conjugate these intermediates with glutathione or sugar moieties, facilitating detoxification and subsequent compartmentalization (Della et al. 2025). Comparable detoxification mechanisms have been described in *Sorghum bicolor* and *Arabidopsis thaliana*, particularly in response to herbicides targeting PPO, ALS, and ACCase (Adhikari et al. 2020; Parcharidou et al. 2024). In cassava, associations involving genes related to redox metabolism, cellular integrity, and xenobiotic conjugation further support the conservation and functional relevance of these pathways in tropical crops (Santos et al. 2022b; Aghogho et al. 2024).

Additionally, the genes *Manes.09G060900* (chromosome 9) and *Manes.15G083800* (chromosome 15) are associated with protein kinase activity, ATP binding, protein phosphorylation, and methyltransferase-related functions. These genes are associated with functions related to cellular signaling and metabolic regulation, suggesting that these biological processes may contribute to herbicide responses. Protein kinases act as key sensors and signal transducers, activating downstream pathways that regulate the expression of detoxification and defense-related genes (Huang et al. 2019; Song et al. 2025). The interplay between redox signaling and metabolic responses is well established in plants exposed to abiotic stress, where reactive oxygen species (ROS) function as signaling molecules that trigger antioxidant defenses and transporter activation (Liao et al. 2023; Ospina et al. 2024). These results are consistent with the polygenic architecture observed in this GWAS, characterized by multiple loci with small effects, indicating that herbicide tolerance in cassava is controlled by a network of interacting metabolic and signaling pathways (Aghogho et al. 2024; Olayinka et al. 2025).

The functional evidence suggests that cassava responses to herbicide exposure involve the integration of membrane transport, cellular signaling, transcriptional regulation, and metabolic adjustment processes. Genes associated with channel activity, transmembrane transport, protein phosphorylation, methyltransferase activity, and RNA processing indicate that tolerance is mediated by coordinated regulatory networks that contribute to cellular homeostasis under chemical stress. These mechanisms are consistent with metabolic resistance pathways widely described across plant species, including xenobiotic transport, signaling cascades, and stress-response regulation (Gaines et al. 2020). Thus, the present GWAS supports that herbicide tolerance in cassava is a quantitative, polygenic trait governed by multiple genes of small effect whose interactions determine the phenotypic response. This reinforces the need for high-resolution genomic approaches and large diversity panels, as highlighted by Aghogho et al. (2024) and Olayinka et al. (2025). A deeper understanding of these pathways provides a valuable foundation for cassava breeding strategies and marker-assisted selection aimed at improving detoxification capacity and enhancing physiological resilience to herbicide stress.

### Comparison with previous genomic studies in cassava

The results of this study are consistent with recent evidence highlighting the genetic complexity of adaptive traits in cassava. Rabbi et al. (2022) and Aghogho et al. (2024) identified multiple QTLs controlling root quality, yield, and tolerance to biotic and abiotic stresses, many of which are located in genomic regions overlapping those identified here. The recurrence of associations in regions harboring genes related to defense responses, secondary metabolism, and cellular growth regulation supports the hypothesis that herbicide tolerance in cassava is governed by a broad regulatory network rather than by single major-effect genes.

The overlap of associated genomic regions on chromosomes 1, 4, and 5, observed both in this study and in Olayinka et al. (2025) suggests the presence of multifunctional genomic regions in cassava. These authors identified significant SNPs on the same chromosomes exhibiting pleiotropic effects and QTL colocalization for traits related to plant architecture and productivity. Similar patterns have been documented in crops such as soybean and rice. Jo et al. (2023) reported a locus on soybean chromosome 5 associated with tolerance to the herbicide bentazon, containing genes involved in detoxification pathways, including ABC transporters and UDP-glycosyltransferases (UGTs). Similarly, Xu et al. (2024) identified loci on rice chromosomes 6, 8, and 10 associated with xenobiotic metabolism and herbicide resistance. These parallels suggest that specific genomic regions may harbor evolutionarily conserved key genes involved in responses to both biotic and chemical stresses, highlighting shared molecular mechanisms underlying plant adaptation.

### Implications for cassava breeding

The genomic regions associated with herbicide response identified in this study, including chr9:1858429 for S-metolachlor and chr4:1751307 for mesotrione, represent promising candidate loci for future validation in cassava breeding programs. Following independent validation, estimation of allele effects, and functional characterization, these loci may support the development of diagnostic markers for marker-assisted selection (MAS) and facilitate the introgression of favorable alleles into breeding populations.

Haplotype-based analyses and block selection strategies have been successfully applied in cassava breeding (Aghogho et al. 2024). Genomic regions harboring multiple associated SNPs, such as those identified on chromosome 5, should be prioritized for fine mapping and haplotype characterization to better resolve potential causal variants. The identification of favorable haplotypes supports QTL pyramiding strategies, allowing the accumulation of beneficial alleles across loci and enhancing tolerance in an additive manner.

Given the polygenic nature of herbicide tolerance, genomic selection (GS) represents a complementary and scalable approach for cassava improvement. GWAS-derived markers can be integrated into GS frameworks in two main ways: (i) by incorporating significant SNPs as fixed effects to improve predictive accuracy, and (ii) by optimizing training populations through the inclusion of genotypes carrying favorable alleles, thereby enhancing genomic estimated breeding values (GEBVs). Previous studies in cassava and other crops have demonstrated consistent gains in prediction accuracy using such strategies, and the integration of GWAS and GS has proven effective in accelerating genetic gain (Esuma et al. 2021).

Although the incorporation of molecular tools into breeding pipelines requires initial investments in genotyping infrastructure and analytical capacity, it offers substantial medium- to long-term benefits by reducing dependence on large-scale phenotyping. In cassava, the development of herbicide-tolerant varieties could enable more efficient weed management, lower production costs, and improve yield stability, particularly for smallholder farmers. These benefits should be further quantified through targeted economic and impact assessments.

## Conclusions

This genome-wide association study identified genomic regions associated with tolerance to mesotrione and S-metolachlor in cassava and revealed substantial genetic variation among the evaluated genotypes. In contrast, no significant associations were detected for chloransulam-methyl under the conditions evaluated, indicating that additional studies using larger populations, higher-density marker platforms, and complementary mapping approaches will be required to clarify its genetic architecture.

For S-metolachlor, a single SNP on chromosome 9 (chr9:1858429) was consistently detected by the BLINK model across both growing seasons and the combined analysis. Although this association represents a reproducible statistical signal, its low minor allele frequency and lack of support from alternative GWAS models indicate that independent validation and functional characterization are required before its biological significance can be established. In contrast, mesotrione tolerance was associated with multiple genomic regions distributed across chromosomes 1, 4, 5, 7, 11, 14, 15, and 18, supporting a complex quantitative genetic architecture.

Candidate genes located near significant SNPs were annotated with functions related to membrane transport, channel activity, cellular signaling, protein phosphorylation, transcriptional regulation, RNA processing, and metabolic regulation. These annotations provide hypotheses regarding biological processes that may contribute to herbicide responses in cassava but require experimental validation.

The distribution of multiple loci across the genome indicates that herbicide tolerance in cassava is a quantitative trait likely controlled by numerous genomic regions with small effects. The genomic regions identified in this study, particularly those associated with mesotrione and the chromosome 9 locus detected for S-metolachlor, provide valuable targets for future fine mapping, functional genomics, and validation studies. Following independent validation, these loci may contribute to marker-assisted and genomic selection strategies aimed at developing cassava cultivars with improved herbicide tolerance.

## Supplementary Information

Below is the link to the electronic supplementary material.

Supplementary Material 1

## Data Availability

The data that support the findings of this study are available from the corresponding author upon reasonable request.
