# Supplementary material for: Pinpointing genomic regions conferring herbicide tolerance in cassava via genome-wide association mapping
Source: Plant Mol Biol. 2026 Sep 9;116(5):95. doi: 10.1007/s11103-026-01761-3 (PMC13558369; doi:10.1007/s11103-026-01761-3)
Supplement: Supplementary file 1 [file 11103_2026_1761_MOESM1_ESM.docx]

**Table S1.** List of cassava genotypes with their geographic and genetic origin, collection state, and date of entry into the cassava germplasm bank at Embrapa Cassava and Fruits.

| **Genotype** | **Country** | **State** | **Genetic origin** | **Date of entry** |
| --- | --- | --- | --- | --- |
| BGM-0004 | Brazil | Bahia | Landrace | 1962 |
| BGM-0005 | Brazil | Minas Gerais | Landrace | 1967 |
| BGM-0011 | Brazil | Bahia | Improved variety | 1965 |
| BGM-0014 | Brazil | Bahia | Improved variety | 1965 |
| BGM-0018 | Brazil | n.i. | Landrace | 1971 |
| BGM-0020 | Brazil | São Paulo | Improved variety | 1967 |
| BGM-0021 | Brazil | Amazonas | Landrace | 1972 |
| BGM-0022 | Brazil | n.i. | Landrace | 1972 |
| BGM-0024 | Brazil | Sergipe | Landrace | 1969 |
| BGM-0025 | Brazil | Bahia | Landrace | 1969 |
| BGM-0029 | Brazil | Bahia | Landrace | 1972 |
| BGM-0030 | Brazil | n.i. | Landrace | 1972 |
| BGM-0036 | Brazil | Bahia | Landrace | 1971 |
| BGM-0040 | Brazil | Bahia | Landrace | 1972 |
| BGM-0046 | Brazil | São Paulo | Improved variety | 1971 |
| BGM-0050 | Brazil | Bahia | Landrace | 1966 |
| BGM-0074 | Brazil | Bahia | Landrace | 1972 |
| BGM-0080 | Brazil | Pernambuco | Landrace | 1969 |
| BGM-0083 | Brazil | Bahia | Landrace | 1969 |
| BGM-0089 | Brazil | n.i. | Landrace | 1973 |
| BGM-0093 | Brazil | n.i. | Landrace | 1973 |
| BGM-0097 | Brazil | Bahia | Improved variety | 1972 |
| BGM-0100 | Brazil | Bahia | Improved variety | 1972 |
| BGM-0106 | Brazil | Sergipe | Landrace | 1972 |
| BGM-0124 | Brazil | n.i. | Landrace | 1972 |
| BGM-0126 | Brazil | Bahia | Landrace | 1972 |
| BGM-0128 | Brazil | Minas Gerais | Landrace | 1972 |
| BGM-0137 | Brazil | Bahia | Landrace | 1972 |
| BGM-0138 | Brazil | Bahia | Landrace | 1972 |
| BGM-0150 | Brazil | Bahia | Landrace | 1972 |
| BGM-0151 | Brazil | Bahia | Landrace | 1972 |
| BGM-0152 | Brazil | Bahia | Landrace | 1972 |
| BGM-0155 | Brazil | Bahia | Landrace | 1972 |
| BGM-0162 | Brazil | Bahia | Landrace | 1977 |
| BGM-0163 | Brazil | Bahia | Landrace | 1972 |
| BGM-0164 | Brazil | Bahia | Landrace | 1969 |
| BGM-0178 | Brazil | Bahia | Landrace | 1972 |
| BGM-0184 | Brazil | Bahia | Landrace | 1969 |
| BGM-0186 | Brazil | Bahia | Landrace | 1972 |
| BGM-0202 | Brazil | Bahia | Landrace | 1971 |
| BGM-0207 | Brazil | Bahia | Improved variety | 1974 |
| BGM-0208 | Brazil | Bahia | Landrace | 1972 |
| BGM-0220 | Brazil | Bahia | Improved variety | 1972 |
| BGM-0226 | Brazil | Bahia | Landrace | 1972 |
| BGM-0235 | Brazil | São Paulo | Improved variety | 1974 |
| BGM-0236 | Brazil | São Paulo | Improved variety | 1974 |
| BGM-0240 | Brazil | Bahia | Improved variety | 1974 |
| BGM-0247 | Brazil | n.i. | Landrace | 1974 |
| BGM-0254 | Brazil | Bahia | Landrace | 1971 |
| BGM-0264 | Brazil | Bahia | Landrace | 1972 |
| BGM-0266 | Brazil | Bahia | Landrace | 1972 |
| BGM-0284 | Brazil | Bahia | Landrace | 1972 |
| BGM-0286 | Brazil | n.i. | Landrace | 1972 |
| BGM-0289 | Colombia | Valle | Improved variety | 1976 |
| BGM-0297 | Colombia | Valle | Improved variety | 1976 |
| BGM-0298 | Colombia | Valle | Improved variety | 1976 |
| BGM-0358 | Brazil | Bahia | Landrace | 1977 |
| BGM-0360 | Colombia | Valle | Improved variety | 1977 |
| BGM-0366 | Brazil | n.i. | Landrace | 1977 |
| BGM-0369 | Brazil | Ceará | Landrace | 1977 |
| BGM-0384 | Brazil | Ceará | Landrace | 1977 |
| BGM-0386 | Brazil | Ceará | Landrace | 1977 |
| BGM-0396 | Brazil | Espírito Santo | Landrace | 1977 |
| BGM-0406 | Brazil | Espírito Santo | Landrace | 1977 |
| BGM-0421 | Brazil | São Paulo | Improved variety | 1977 |
| BGM-0426 | Brazil | n.i. | Improved variety | 1977 |
| BGM-0437 | Brazil | n.i. | Landrace | 1977 |
| BGM-0439 | Brazil | n.i. | Landrace | 1977 |
| BGM-0441 | Brazil | Rio Grande do Sul | Landrace | 1977 |
| BGM-0471 | Brazil | n.i. | Landrace | 1977 |
| BGM-0476 | Brazil | n.i. | Landrace | 1977 |
| BGM-0494 | Brazil | Rio de Janeiro | Landrace | 1977 |
| BGM-0496 | Brazil | Espírito Santo | Landrace | 1977 |
| BGM-0514 | Brazil | n.i. | Improved variety | 1977 |
| BGM-0517 | Brazil | Minas Gerais | Landrace | 1977 |
| BGM-0520 | Brazil | Minas Gerais | Landrace | 1977 |
| BGM-0521 | Brazil | Rio de Janeiro | Improved variety | 1977 |
| BGM-0523 | Brazil | Minas Gerais | Landrace | 1977 |
| BGM-0529 | Brazil | n.i. | Landrace | 1977 |
| BGM-0547 | Brazil | Pernambuco | Landrace | 1978 |
| BGM-0550 | Brazil | Bahia | Landrace | 1978 |
| BGM-0551 | Brazil | Pará | Improved variety | 1978 |
| BGM-0556 | Brazil | Bahia | Landrace | 1978 |
| BGM-0562 | Brazil | Bahia | Landrace | 1978 |
| BGM-0573 | Brazil | Paraná | Landrace | 1978 |
| BGM-0587 | Brazil | Pernambuco | Landrace | 1978 |
| BGM-0589 | Brazil | n.i. | Landrace | 1978 |
| BGM-0592 | Brazil | Rio Grande do Sul | Landrace | 1978 |
| BGM-0608 | Brazil | n.i. | Landrace | 1980 |
| BGM-0613 | Brazil | Ceará | Landrace | 1980 |
| BGM-0624 | Brazil | Ceará | Landrace | 1980 |
| BGM-0635 | Brazil | Ceará | Landrace | 1980 |
| BGM-0636 | Brazil | Ceará | Landrace | 1980 |
| BGM-0638 | Brazil | Ceará | Landrace | 1980 |
| BGM-0639 | Brazil | São Paulo | Landrace | 1980 |
| BGM-0649 | Brazil | Ceará | Landrace | 1981 |
| BGM-0662 | Brazil | Maranhão | Landrace | 1981 |
| BGM-0665 | Brazil | Maranhão | Landrace | 1981 |
| BGM-0679 | Brazil | Maranhão | Landrace | 1981 |
| BGM-0680 | Brazil | Maranhão | Landrace | 1981 |
| BGM-0700 | Brazil | Maranhão | Landrace | 1981 |
| BGM-0714 | Brazil | Bahia | Landrace | 1981 |
| BGM-0737 | Brazil | Espírito Santo | Landrace | 1981 |
| BGM-0790 | Brazil | Bahia | Landrace | 1981 |
| BGM-0810 | Brazil | Alagoas | Landrace | 1981 |
| BGM-0820 | Brazil | Sergipe | Landrace | 1981 |
| BGM-0822 | Brazil | Sergipe | Landrace | 1972 |
| BGM-0831 | Brazil | Sergipe | Landrace | 1981 |
| BGM-0842 | Brazil | Sergipe | Landrace | 1981 |
| BGM-0843 | Brazil | Sergipe | Landrace | 1981 |
| BGM-0857 | Brazil | Sergipe | Landrace | 1981 |
| BGM-0879 | Brazil | Santa Catarina | Landrace | 1982 |
| BGM-0882 | Colombia | Valle | Improved variety | 1982 |
| BGM-0884 | Colombia | Valle | Improved variety | 1982 |
| BGM-0888 | Brazil | São Paulo | Improved variety | 1982 |
| BGM-0889 | Brazil | Tocantins | Landrace | 1982 |
| BGM-0894 | Colombia | Valle | Improved variety | 1982 |
| BGM-0901 | Brazil | Ceará | Landrace | 1982 |
| BGM-0906 | Colombia | Valle | Improved variety | 1987 |
| BGM-0914 | Brazil | Pará | Landrace | 1987 |
| BGM-0962 | Brazil | Amazonas | Landrace | 1987 |
| BGM-0965 | Brazil | Amazonas | Landrace | 1987 |
| BGM-0977 | Brazil | Amazonas | Landrace | 1987 |
| BGM-0981 | Brazil | Amazonas | Landrace | 1987 |
| BGM-0984 | Brazil | Amazonas | Landrace | 1987 |
| BGM-0987 | Brazil | Amazonas | Landrace | 1987 |
| BGM-0992 | Brazil | Amazonas | Landrace | 1987 |
| BGM-1010 | Brazil | n.i. | Landrace | 1988 |
| BGM-1026 | Brazil | Bahia | Landrace | 1987 |
| BGM-1028 | Brazil | Bahia | Landrace | 1987 |
| BGM-1105 | Brazil | Bahia | Landrace | 1987 |
| BGM-1123 | Brazil | Bahia | Landrace | 1987 |
| BGM-1144 | Brazil | Amazonas | Landrace | 1988 |
| BGM-1150 | Brazil | Bahia | Landrace | 1989 |
| BGM-1168 | Brazil | n.i. | Landrace | 1988 |
| BGM-1171 | Brazil | Pará | Landrace | 1988 |
| BGM-1190 | Brazil | Pará | Landrace | 1989 |
| BGM-1214 | Brazil | Pará | Landrace | 1989 |
| BGM-1227 | Brazil | n.i. | Landrace | 1990 |
| BGM-1251 | Nigeria | n.i. | Improved variety | 1989 |
| BGM-1268 | Brazil | Pernambuco | Landrace | 1991 |
| BGM-1269 | Brazil | Pernambuco | Landrace | 1991 |
| BGM-1270 | Brazil | Pernambuco | Landrace | 1991 |
| BGM-1274 | Brazil | Pernambuco | Landrace | 1991 |
| BGM-1313 | Brazil | Pernambuco | Landrace | 1991 |
| BGM-1321 | Brazil | Pernambuco | Landrace | 1991 |
| BGM-1326 | Brazil | Pernambuco | Landrace | 1991 |
| BGM-1331 | Brazil | Paraíba | Landrace | 1991 |
| BGM-1335 | Brazil | Pernambuco | Landrace | 1991 |
| BGM-1349 | Brazil | Paraíba | Landrace | 1991 |
| BGM-1362 | Brazil | Paraíba | Landrace | 1991 |
| BGM-1363 | Brazil | Paraíba | Landrace | 1991 |
| BGM-1368 | Brazil | Paraíba | Landrace | 1991 |
| BGM-1385 | Brazil | Rio Grande do Norte | Landrace | 1991 |
| BGM-1389 | Brazil | Rio Grande do Norte | Landrace | 1991 |
| BGM-1406 | Brazil | Rio Grande do Norte | Landrace | 1991 |
| BGM-1441 | Brazil | Bahia | Landrace | 1991 |
| BGM-1456 | Brazil | Mato Grosso | Landrace | 1990 |
| BGM-1508 | Brazil | Bahia | Landrace | 1994 |
| BGM-1521 | Brazil | Bahia | Landrace | 1994 |
| BGM-1545 | Brazil | Bahia | Landrace | 1994 |
| BGM-1583 | Brazil | Piauí | Landrace | 1994 |
| BGM-1588 | Brazil | Piauí | Landrace | 1994 |
| BGM-1604 | Brazil | Piauí | Landrace | 1994 |
| BGM-1606 | Brazil | Piauí | Landrace | 1994 |
| BGM-1614 | Brazil | Piauí | Landrace | 1994 |
| BGM-1621 | Brazil | Paraíba | Landrace | 1995 |
| BGM-1636 | Brazil | Ceará | Landrace | 1995 |
| BGM-1637 | Brazil | Pará | Landrace | 1995 |
| BGM-1640 | Brazil | Bahia | Landrace | 1995 |
| BGM-1646 | Brazil | Sergipe | Landrace | 1996 |
| BGM-1648 | Brazil | Bahia | Landrace | 1996 |
| BGM-1649 | Brazil | Bahia | Landrace | 1996 |
| BGM-1655 | Brazil | Sergipe | Landrace | 1996 |
| BGM-1660 | Brazil | Bahia | Landrace | 1996 |
| BGM-1684 | Brazil | Bahia | Landrace | 1999 |
| BGM-1685 | Brazil | Bahia | Landrace | 1999 |
| BGM-1698 | Brazil | Mato Grosso do Sul | Landrace | 2001 |
| BGM-1704 | Brazil | Amazonas | Improved variety | 2001 |
| BGM-1718 | Brazil | Amazonas | Improved variety | 2001 |
| BGM-1723 | Brazil | Bahia | Improved variety | 2001 |
| BGM-1726 | Brazil | Bahia | Improved variety | 2001 |
| BGM-1733 | Brazil | Maranhão | Landrace | 2001 |
| BGM-1741 | Brazil | Maranhão | Landrace | 2001 |
| BR-11-34-41 | Brazil | Bahia | Improved variety | n.i. |
| BR-11-34-45 | Brazil | Bahia | Improved variety | n.i. |
| BR-11-34-64 | Brazil | Bahia | Improved variety | n.i. |
| BR-11-34-69 | Brazil | Bahia | Improved variety | n.i. |
| BR-14-006-02 | Brazil | Bahia | Improved variety | n.i. |
| BR-14-010-11 | Brazil | Bahia | Improved variety | n.i. |
| BRS-Formosa | Brazil | Bahia | Improved variety | 2002 |
| BRS-Kiriris | Brazil | Bahia | Improved variety | 2001 |
| Cigana | Brazil | Pernambuco | Landrace | 2013 |
| Corrente | Brazil | Bahia | Landrace | 2012 |

**Table S2.** Distribution of SNP markers across the 18 chromosomes of the cassava genome (*Manihot esculenta* Crantz).

| **Chromosome** | **No. of SNPs** | **Maximum position (bp)** | **Length (Mb)** | **Density (SNP/kb)** |
| --- | --- | --- | --- | --- |
| 1 | 2,115 | 34,950,389 | 34.950 | 0.0605 |
| 2 | 1,491 | 32,369,571 | 32.370 | 0.0461 |
| 3 | 1,609 | 29,305,664 | 29.306 | 0.0549 |
| 4 | 1,180 | 28,730,625 | 28.731 | 0.0411 |
| 5 | 1,420 | 28,333,703 | 28.334 | 0.0501 |
| 6 | 1,293 | 27,889,445 | 27.889 | 0.0464 |
| 7 | 862 | 26,784,510 | 26.785 | 0.0322 |
| 8 | 1,130 | 33,846,080 | 33.846 | 0.0334 |
| 9 | 1,215 | 29,403,154 | 29.403 | 0.0413 |
| 10 | 1,360 | 26,316,398 | 26.316 | 0.0517 |
| 11 | 1,349 | 27,265,643 | 27.266 | 0.0495 |
| 12 | 920 | 31,557,124 | 31.557 | 0.0292 |
| 13 | 1,021 | 28,097,619 | 28.098 | 0.0363 |
| 14 | 1,362 | 24,504,648 | 24.505 | 0.0556 |
| 15 | 1,307 | 26,009,214 | 26.009 | 0.0503 |
| 16 | 955 | 28,955,996 | 28.956 | 0.0330 |
| 17 | 1,384 | 27,358,816 | 27.359 | 0.0506 |
| 18 | 1,074 | 25,097,165 | 25.097 | 0.0428 |
| **Total** | **23,047** | — | **532.495** | **0.0433** |

**Table S3**. Genomic inflation factors (λGC) estimated for each GWAS analysis according to herbicide, analytical scenario, and GWAS model.

| **Herbicide** | **Dataset** | **GWAS model** | **λGC** |
| --- | --- | --- | --- |
| Mesotrione | 2024 | BLINK | 1.063 |
| Mesotrione | 2024 | MLM | 0.725 |
| Mesotrione | 2024 | MLMM | 1.070 |
| Mesotrione | Combined | BLINK | 0.828 |
| Mesotrione | Combined | MLM | 0.623 |
| Mesotrione | Combined | MLMM | 0.671 |
| S-metolachlor | 2023 | BLINK | 0.861 |
| S-metolachlor | 2024 | BLINK | 0.850 |
| S-metolachlor | Combined | BLINK | 0.850 |

λGC values close to 1 indicate appropriate model calibration, whereas values below or above 1 indicate conservative or inflated test statistics, respectively.

**Table S4.** Summary of SNPs detected across GWAS models and datasets for mesotrione and S-metolachlor.

| **SNP** | **Chr** | **Position (bp)** | **Herbicide** | **Dataset** | **GWAS model** | **−log10(P)** | **Support classᵃ** |
| --- | --- | --- | --- | --- | --- | --- | --- |
| chr11:25011154 | 11 | 25011154 | Mesotrione | 2024 | BLINK | 12.501 | MM |
|  |  |  |  |  | MLM | 13.497 |  |
|  |  |  |  |  | MLMM | 64.611 |  |
| chr11:25007496 | 11 | 25007496 | Mesotrione | 2024 | BLINK | 10.852 | MM |
|  |  |  |  |  | MLMM | 52.017 |  |
| chr1:11468003 | 1 | 11468003 | Mesotrione | 2024 | BLINK | 25.644 | MM |
|  |  |  |  |  | MLM | 9.194 |  |
| chr1:24877106 | 1 | 24877106 | Mesotrione | 2024 | BLINK | 16.529 | MM |
|  |  |  |  |  | MLM | 8.826 |  |
| chr1:16899387 | 1 | 16899387 | Mesotrione | 2024 | BLINK | 7.997 | MM |
|  |  |  |  |  | MLM | 12.404 |  |
| chr4:1751307 | 4 | 1751307 | Mesotrione | Combined | BLINK | 7.349 | MM |
|  |  |  |  |  | MLM | 6.564 |  |
|  |  |  |  |  | MLMM | 7.511 |  |
| chr9:1858429 | 9 | 1858429 | S-metolachlor | 2023 | BLINK | 5.823 | SE |
| chr9:1858429 | 9 | 1858429 | S-metolachlor | 2024 | BLINK | 5.778 | SE |
| chr9:1858429 | 9 | 1858429 | S-metolachlor | Combined | BLINK | 5.778 | SE |

ᵃ MM, SNP detected by multiple GWAS models within the same dataset; SE, SNP detected by a single GWAS model within the same dataset.

**Table S5.** Candidate genes identified within genomic regions associated with herbicide tolerance in cassava (*Manihot esculenta* Crantz) based on genome-wide association study (GWAS) results. The table summarizes significant single nucleotide polymorphisms (SNPs), their chromosomal locations and genomic positions, associated candidate genes, and corresponding functional annotations derived from Gene Ontology (GO) terms. The predicted biological functions indicate that these genes are involved in key processes such as xenobiotic detoxification and transport, cellular signaling, oxidative stress response, and metabolic regulation, supporting their potential role in the physiological and structural mechanisms underlying herbicide tolerance in cassava.

| **Chromosome** | **Downstream (kb)** | **Upstream (kb)** | **SNP** | **Pos 1** | **Pos 2** | **Candidate gene** | **GO** |
| --- | --- | --- | --- | --- | --- | --- | --- |
| 1 | 24857106 | 24897106 | chr1:24877106 | 24859033 | 24860928 | Manes.01G088600; Manes.01G088700; Manes.01G088800 | RNA binding/RNA processing/RNA methyltransferase activity; serine-type endopeptidase activity/proteolysis/serine-type peptidase activity |
| 7 | 761893 | 801893 | chr7:781893 | 762711 | 765796 | Manes.07G005800; Manes.07G005900; Manes.07G006000; Manes.07G006200; Manes.07G006300 | antiporter activity/membrane/xenobiotic transmembrane transporter activity/transmembrane transport; protein binding/metal ion binding |
| 15 | 3237801 | 3277801 | chr15:3257801 | 3239077 | 3239854 | Manes.15G039600; Manes.15G039700; Manes.15G039800; Manes.15G040000; Manes.15G040100; Manes.15G040200; Manes.15G040300 | DNA binding/DNA-binding transcription factor activity/regulation of DNA-templated transcription; transmembrane transporter activity/transmembrane transport |
| 11 | 24991154 | 25031154 | chr11:25011154 | 24996487 | 25012064 | Manes.11G107000; Manes.11G107050; Manes.11G107101 | monooxygenase activity/iron ion binding/oxidoreductase activity, acting on paired donors, with incorporation or reduction of molecular oxygen/heme binding |
| 11 | 24987496 | 25027496 | chr11:25007496 | 24973987 | 24989021 | Manes.11G106900; Manes.11G107000; Manes.11G107050 | acyltransferase activity; monooxygenase activity/iron ion binding/oxidoreductase activity, acting on paired donors, with incorporation or reduction of molecular oxygen/heme binding |
| 18 | 8649973 | 8689973 | chr18:8669973 | 8660365 | 8662375 | Manes.18G074748; Manes.18G074760; Manes.18G075300; Manes.18G075400; Manes.18G075500; Manes.18G075600 | DNA binding/DNA-binding transcription factor activity/regulation of DNA-templated transcription; transsulfuration/pyridoxal phosphate binding |
| 11 | 24991189 | 25031189 | chr11:25011189 | 24996487 | 25012064 | Manes.11G107000; Manes.11G107050; Manes.11G107101 | monooxygenase activity/iron ion binding/oxidoreductase activity, acting on paired donors, with incorporation or reduction of molecular oxygen/heme binding |
| 3 | 2148588 | 2188588 | chr3:2168588 | 2145748 | 2152617 | Manes.03G026700; Manes.03G026800; Manes.03G026901; Manes.03G027000; Manes.03G027100 | intracellular glucose homeostasis/hexokinase activity/ATP binding/D-glucose binding/carbohydrate metabolic process/phosphotransferase activity, alcohol group as acceptor; protein kinase activity/protein tyrosine kinase activity/protein binding/ATP binding/protein phosphorylation |
| 18 | 9215000 | 9255000 | chr18:9235000 | 9217853 | 9218986 | Manes.18G079700; Manes.18G079800; Manes.18G079900; Manes.18G080000; Manes.18G080100; Manes.18G080200 | hydrolase activity, hydrolyzing O-glycosyl compounds/carbohydrate metabolic process; membrane/metal ion transport/metal ion transmembrane transporter activity; zinc ion binding |
| 18 | 9215026 | 9255026 | chr18:9235026 | 9217853 | 9218986 | Manes.18G079700; Manes.18G079800; Manes.18G079900; Manes.18G080000; Manes.18G080100; Manes.18G080200 | hydrolase activity, hydrolyzing O-glycosyl compounds/carbohydrate metabolic process; membrane/metal ion transport/metal ion transmembrane transporter activity; zinc ion binding |
| 6 | 25076573 | 25116573 | chr6:25096573 | 25081731 | 25083026 | Manes.06G115440; Manes.06G115460; Manes.06G115480; Manes.06G115500 | nucleoside diphosphate kinase activity/GTP biosynthetic process/UTP biosynthetic process/CTP biosynthetic process |
| 6 | 25076588 | 25116588 | chr6:25096588 | 25081731 | 25083026 | Manes.06G115440; Manes.06G115460; Manes.06G115480; Manes.06G115500 | nucleoside diphosphate kinase activity/GTP biosynthetic process/UTP biosynthetic process/CTP biosynthetic process |
| 6 | 25076622 | 25116622 | chr6:25096622 | 25081731 | 25083026 | Manes.06G115440; Manes.06G115460; Manes.06G115480; Manes.06G115500 | nucleoside diphosphate kinase activity/GTP biosynthetic process/UTP biosynthetic process/CTP biosynthetic process |
| 13 | 24574495 | 24614495 | chr13:24594495 | 24590296 | 24596127 | Manes.13G096000 |  |
| 6 | 26591303 | 26631303 | chr6:26611303 | 26588116 | 26592088 | Manes.06G132000; Manes.06G132100; Manes.06G132200; Manes.06G132300; Manes.06G132400; Manes.06G132500; Manes.06G132600; Manes.06G132700 | hydrolase activity; kinase activity; membrane/transmembrane transporter activity/transmembrane transport; protein kinase activity/protein tyrosine kinase activity/ubiquitin-protein transferase activity/ATP binding/protein phosphorylation/protein ubiquitination |
| 9 | 21854919 | 21894919 | chr9:21874919 | 21864373 | 21867681 | Manes.09G077900 | hydrolase activity, hydrolyzing O-glycosyl compounds/carbohydrate metabolic process |
| 10 | 22960602 | 23000602 | chr10:22980602 | 22960101 | 22964900 | Manes.10G106400; Manes.10G106500; Manes.10G106700 | RNA binding/nucleus/cytoplasm/mRNA processing; membrane/transmembrane transporter activity/transmembrane transport |
| 1 | 7440013 | 7480013 | chr1:7460013 | 7455805 | 7458309 | Manes.01G037300; Manes.01G037400; Manes.01G037500; Manes.01G038033; Manes.01G038566 | intracellular protein transport; protein binding |
| 1 | 7440086 | 7480086 | chr1:7460086 | 7455805 | 7458309 | Manes.01G037300; Manes.01G037400; Manes.01G037500; Manes.01G038033; Manes.01G038566 | intracellular protein transport; protein binding |
| 13 | 2442717 | 2482717 | chr13:2462717 | 2452383 | 2454500 | Manes.13G024000; Manes.13G024100; Manes.13G024200; Manes.13G024300; Manes.13G024400; Manes.13G024500 | protein binding |
| 13 | 2442732 | 2482732 | chr13:2462732 | 2452383 | 2454500 | Manes.13G024000; Manes.13G024100; Manes.13G024200; Manes.13G024300; Manes.13G024400; Manes.13G024500 | protein binding |
| 15 | 21703327 | 21743327 | chr15:21723327 | 21718381 | 21723510 | Manes.15G180800 |  |
| 4 | 2995128 | 3035128 | chr4:3015128 | 3006067 | 3010969 | Manes.04G025500; Manes.04G025600; Manes.04G025700 | phosphatidylinositol-4,5-bisphosphate phospholipase C activity/lipid metabolic process/signal transduction/phosphoric diester hydrolase activity/intracellular signal transduction; phospholipid transporter activity/phospholipid transport |
| 3 | 2900362 | 2940362 | chr3:2920362 | 2928078 | 2933150 | Manes.03G034400 | translation initiation factor activity/translational initiation |
| 15 | 6444755 | 6484755 | chr15:6464755 | 6443547 | 6445942 | Manes.15G081401; Manes.15G081500; Manes.15G081600 | DNA-binding transcription factor activity |
| 15 | 6667927 | 6707927 | chr15:6687927 | 6666028 | 6668459 | Manes.15G083800; Manes.15G083900; Manes.15G084000; Manes.15G084100; Manes.15G084200; Manes.15G084300; Manes.15G084400; Manes.15G084500; Manes.15G084600; Manes.15G084650 | ADP binding; S-adenosylmethionine-dependent methyltransferase activity; membrane; protein kinase activity/protein tyrosine kinase activity/ATP binding/protein phosphorylation |
| 15 | 6709375 | 6749375 | chr15:6729375 | 6710973 | 6713373 | Manes.15G084700; Manes.15G084800; Manes.15G084900; Manes.15G085001; Manes.15G085100; Manes.15G085200 | DNA binding/regulation of DNA-templated transcription; signal transduction |
| 15 | 6709388 | 6749388 | chr15:6729388 | 6710973 | 6713373 | Manes.15G084700; Manes.15G084800; Manes.15G084900; Manes.15G085001; Manes.15G085100; Manes.15G085200 | DNA binding/regulation of DNA-templated transcription; signal transduction |
| 15 | 6709423 | 6749423 | chr15:6729423 | 6710973 | 6713373 | Manes.15G084700; Manes.15G084800; Manes.15G084900; Manes.15G085001; Manes.15G085100; Manes.15G085200 | DNA binding/regulation of DNA-templated transcription; signal transduction |
| 11 | 9227987 | 9267987 | chr11:9247987 | 9240863 | 9241162 | Manes.11G068114; Manes.11G068128; Manes.11G068142; Manes.11G068156; Manes.11G068170 | response to auxin |
| 3 | 1920615 | 1960615 | chr3:1940615 | 1928826 | 1929886 | Manes.03G023500; Manes.03G023600; Manes.03G023700; Manes.03G023800; Manes.03G023900; Manes.03G024000 | DNA binding/DNA-directed RNA polymerase activity/DNA-templated transcription; antioxidant activity/oxidoreductase activity; methyltransferase activity; protein binding; protein dimerization activity |
| 13 | 26629457 | 26669457 | chr13:26649457 | 26637347 | 26639559 | Manes.13G103533; Manes.13G103666 | DNA binding/DNA-binding transcription factor activity/nucleus/regulation of DNA-templated transcription/protein dimerization activity |
| 18 | 11640959 | 11680959 | chr18:11660959 | 11650956 | 11652996 | Manes.18G101800; Manes.18G101900; Manes.18G101975 | magnesium ion binding/terpene synthase activity/lyase activity |
| 13 | 3326288 | 3366288 | chr13:3346288 | 3325719 | 3327418 | Manes.13G033600; Manes.13G033700; Manes.13G033800; Manes.13G033900; Manes.13G034000; Manes.13G034100; Manes.13G034200; Manes.13G034300; Manes.13G034400 | actin binding; histone acetyltransferase complex/obsolete histone acetylation; nutrient reservoir activity |
| 3 | 1302168 | 1342168 | chr3:1322168 | 1307483 | 1312167 | Manes.03G014900; Manes.03G015000; Manes.03G015100; Manes.03G015200; Manes.03G015400 | DNA binding/DNA-binding transcription factor activity/regulation of DNA-templated transcription; protein binding |
| 3 | 1327606 | 1367606 | chr3:1347606 | 1326406 | 1335627 | Manes.03G015200; Manes.03G015400; Manes.03G015500; Manes.03G015600; Manes.03G015700; Manes.03G015800 | DNA binding/DNA-binding transcription factor activity/regulation of DNA-templated transcription; DNA binding/RNA helicase activity/ATP binding/hydrolase activity; structural constituent of ribosome/ribosome/translation/rRNA binding |
| 3 | 1412228 | 1452228 | chr3:1432228 | 1412075 | 1415306 | Manes.03G016800; Manes.03G016900; Manes.03G017000; Manes.03G017100; Manes.03G017200 | DNA binding |
| 3 | 1565173 | 1605173 | chr3:1585173 | 1564556 | 1573949 | Manes.03G018600; Manes.03G018700; Manes.03G018800; Manes.03G018900; Manes.03G019000; Manes.03G019100 | ATP binding/membrane/ATP hydrolysis activity/ABC-type transporter activity; ATP binding/mismatch repair/mismatched DNA binding; guanyl-nucleotide exchange factor activity/regulation of ARF protein signal transduction |
| 2 | 12558437 | 12598437 | chr2:12578437 | 12557686 | 12558876 | Manes.02G148600; Manes.02G148650; Manes.02G148700; Manes.02G148800; Manes.02G148900; Manes.02G149000; Manes.02G149100 | DNA binding/regulation of DNA-templated transcription/sequence-specific DNA binding; electron transfer activity; tRNA modification/pentosyltransferase activity; zinc ion binding |
| 11 | 3921709 | 3961709 | chr11:3941709 | 3921861 | 3929285 | Manes.11G039800; Manes.11G039864; Manes.11G039928 | protein kinase activity/protein tyrosine kinase activity/ATP binding/protein phosphorylation/protein catabolic process |
| 13 | 3341391 | 3381391 | chr13:3361391 | 3340353 | 3341561 | Manes.13G033800; Manes.13G033900; Manes.13G034000; Manes.13G034100; Manes.13G034200; Manes.13G034300; Manes.13G034400 | nutrient reservoir activity |
| 13 | 3341392 | 3381392 | chr13:3361392 | 3340353 | 3341561 | Manes.13G033800; Manes.13G033900; Manes.13G034000; Manes.13G034100; Manes.13G034200; Manes.13G034300; Manes.13G034400 | nutrient reservoir activity |
| 13 | 3341397 | 3381397 | chr13:3361397 | 3340353 | 3341561 | Manes.13G033800; Manes.13G033900; Manes.13G034000; Manes.13G034100; Manes.13G034200; Manes.13G034300; Manes.13G034400 | nutrient reservoir activity |
| 12 | 4568097 | 4608097 | chr12:4588097 | 4573875 | 4577903 | Manes.12G038900; Manes.12G039000; Manes.12G039100; Manes.12G039200; Manes.12G039300 | ammonium channel activity/membrane/ammonium transmembrane transport; oxidoreductase activity; protein binding |
| 9 | 9865513 | 9905513 | chr9:9885513 | 9895058 | 9898018 | Manes.09G060900; Manes.09G061000 | protein kinase activity/protein tyrosine kinase activity/protein binding/ATP binding/protein phosphorylation |
| 14 | 7295377 | 7335377 | chr14:7315377 | 7298696 | 7304234 | Manes.14G082800; Manes.14G082900; Manes.14G083000; Manes.14G083100; Manes.14G083200; Manes.14G083400; Manes.14G083500 | calmodulin binding; oxidoreductase activity/flavin adenine dinucleotide binding/NADP binding; protein dimerization activity |
| 10 | 2854258 | 2894258 | chr10:2874258 | 2888465 | 2896172 | Manes.10G028501 | ATP hydrolysis activity/ADP binding |
| 6 | 24908379 | 24948379 | chr6:24928379 | 24907330 | 24913840 | Manes.06G113700; Manes.06G113800; Manes.06G113900; Manes.06G114000; Manes.06G114100; Manes.06G114200; Manes.06G114300 | S-adenosylmethionine-dependent methyltransferase activity; metal ion binding; protein binding |
| 5 | 3876743 | 3916743 | chr5:3896743 | 3868020 | 3878029 | Manes.05G049400; Manes.05G049500; Manes.05G049600 | nucleotidyltransferase activity |
| 5 | 4243587 | 4283587 | chr5:4263587 | 4239239 | 4244799 | Manes.05G053500; Manes.05G053600; Manes.05G053700; Manes.05G053800; Manes.05G053900; Manes.05G054000; Manes.05G054050; Manes.05G054100 | lipid transport/lipid binding; nucleotide binding/transporter activity/ATP binding/membrane/ATP hydrolysis activity |
| 5 | 4243683 | 4283683 | chr5:4263683 | 4239239 | 4244799 | Manes.05G053500; Manes.05G053600; Manes.05G053700; Manes.05G053800; Manes.05G053900; Manes.05G054000; Manes.05G054050; Manes.05G054100 | lipid transport/lipid binding; nucleotide binding/transporter activity/ATP binding/membrane/ATP hydrolysis activity |
| 5 | 4301487 | 4341487 | chr5:4321487 | 4306205 | 4307225 | Manes.05G054400; Manes.05G054500; Manes.05G054600; Manes.05G054700; Manes.05G054800 | DNA binding/DNA-binding transcription factor activity/regulation of DNA-templated transcription; methyltransferase activity/methylation |
| 5 | 4411525 | 4451525 | chr5:4431525 | 4400091 | 4426193 | Manes.05G055300; Manes.05G055400; Manes.05G055500; Manes.05G055600; Manes.05G055700; Manes.05G055800 | GTPase activity/GTP binding; electron transfer activity; nucleus/RNA splicing/exon-exon junction complex |
| 7 | 1694750 | 1734750 | chr7:1714750 | 1684648 | 1698059 | Manes.07G013200; Manes.07G013300; Manes.07G013400; Manes.07G013500; Manes.07G013600; Manes.07G013700 | enzyme inhibitor activity/pectinesterase activity/cell wall modification |
| 4 | 1731307 | 1771307 | chr4:1751307 | 1729045 | 1739759 | Manes.04G013000; Manes.04G013101 | metal ion binding |
| 14 | 8716846 | 8756846 | chr14:8736846 | 8710557 | 8717288 | Manes.14G100300; Manes.14G100500; Manes.14G100600; Manes.14G100700; Manes.14G100900; Manes.14G101000; Manes.14G101100 | chromatin binding; cytoplasm/intermembrane lipid transfer/lipid transfer activity; membrane/transmembrane transporter activity/transmembrane transport; metal ion binding |
| 2 | 12784185 | 12824185 | chr2:12804185 | 12777177 | 12787534 | Manes.02G151900; Manes.02G152000; Manes.02G152100; Manes.02G152200; Manes.02G152250; Manes.02G152300; Manes.02G152400; Manes.02G152500; Manes.02G152600; Manes.02G152700 | channel activity/membrane/transmembrane transport; signal peptidase complex/signal peptide processing/membrane |
| 9 | 1838429 | 1878429 | chr9:1858429 | 1845368 | 1853325 | Manes.09G008600; Manes.09G008801; Manes.09G009000 | DNA binding/DNA replication; methyltransferase activity |

**Figure S1**. Genome-wide association study (GWAS) results for the area under the phytotoxicity progress curve (AUPPC) of chloransulam-methyl, evaluated in a panel of 194 cassava genotypes. The left panel shows a circular Manhattan plot displaying −log₁₀(p) values across genomic positions. The right panel presents Q–Q plots for the MLM (mixed linear model), MLMM (multi-locus mixed model), and BLINK (Bayesian information and linkage disequilibrium iteratively nested keyway) models, illustrating model fit and genomic inflation.

**Figure S2**. Genome-wide association study (GWAS) results for the percentage reduction of accumulated phytotoxic symptoms over time (PctReduction) following chloransulam-methyl application, evaluated in 194 cassava genotypes. The left panel shows a circular Manhattan plot of −log₁₀(p) values across genomic positions. The right panel displays Q–Q plots for the MLM, MLMM, and BLINK models, highlighting model performance and genomic inflation.

**Figure S3**. Distribution of minor allele frequency (MAF) based on 23,047 SNP markers genotyped across 194 cassava genotypes.
